# Current treatment of lupus nephritis: an overview of the new guidelines

**DOI:** 10.1590/2175-8239-JBN-2025-0092en

**Published:** 2025-10-13

**Authors:** Gabriel Teixeira Montezuma Sales, Natália Janoni Macedo, Edgard Torres dos Reis, Gianna Mastroianni Kirsztajn

**Affiliations:** 1 Universidade Federal de São Paulo, Escola Paulista de Medicina, Departamento de Medicina, São Paulo, SP, Brazil.

**Keywords:** Lupus Nephritis, Systemic Lupus Erythematosus, Guidelines, KDIGO, Immunosuppression, Glomerulopathies, Therapeutic Targets

## Abstract

**Introduction:** Given the relevance of renal involvement in systemic lupus erythematosus (SLE) and new approaches to the disease and its treatment, this article aimed to synthesize the main updates in the diagnosis, management, and treatment of lupus nephritis (LN), based on recent publications of international reference guidelines in nephrology and rheumatology, in addition to highlighting aspects of interest from the 2024 national guidelines of the Brazilian Society of Rheumatology (SBR). The treatments for each class of lupus nephritis are described, as well as the therapeutic targets, underlining similarities and differences between the guidelines. In general, they recommend that induction (“initial”) treatment of proliferative classes be performed with monotherapy using mycophenolate or intravenous cyclophosphamide, or with multitarget regimens, using corticosteroids, mycophenolate or cyclophosphamide, and a calcineurin inhibitor or belimumab as a third drug. A change in therapy should be considered if the expected response target is not achieved, which presents subtle differences among current consensus guidelines. Maintenance (“subsequent”) treatment should preferably be performed with mycophenolate, azathioprine, or multi-target therapies. Emerging scientific evidence has provided treatment options that impact the management of lupus nephritis, thereby justifying the publication of new guidelines in recent months. Critically analyzing these guidelines may assist in decision-making for the individualized treatment of individuals with this disease.

## Introduction

Systemic Lupus Erythematosus (SLE) is a chronic autoimmune inflammatory disease whose etiopathogenesis involves the interaction between genetic and environmental factors, characterized by wide variability in presentation, clinical course, and severity. In general, it progresses with alternating periods of activity and remission, affecting multiple organs and systems, with the kidney being frequently affected. Renal involvement in SLE may occur in its various compartments: tubulointerstitial, vascular, and glomerular, and may present as different syndromes associated with glomerular diseases (nephrotic and nephritic syndrome, rapidly progressive glomerulonephritis, asymptomatic urinary abnormalities, among others)^1^.

The incidence of lupus nephritis (LN) varies considerably across different regions of the world, races, and ethnicities, affecting 20−60% of people with SLE, depending on the population studied^2,3,4,5^. Renal involvement in SLE, in turn, is associated with increased morbidity and mortality, especially among patients who progress to kidney failure - an event observed in approximately 10−30% of cases - a percentage that may be higher in those with proliferative histological classes^6,7,8,9^.

The primary goal of LN treatment is to achieve complete remission (CR), which is associated with favorable long-term renal prognosis^8,10^. Ten-year renal survival increases from 46% to 95% if remission is achieved^11^. However, despite the available therapeutic regimens, less than 50% of patients achieve CR after the first 6 months of treatment, and among those who do, recurrence occurs in about 25% of cases within 2-3 years^10,12,13,14^.

In this sense, LN treatment has been the subject of numerous studies over the last decade, driven by the development of new technologies and therapeutic options. Of particular note are studies demonstrating the possibility of multitarget therapies as a first-line regimen for induction treatments (“initial,” according to the most recently adopted terminology) in proliferative classes, either with belimumab associated with cyclophosphamide CYC or mycophenolate (MMF), or with calcineurin inhibitors (CNIs), particularly voclosporin, combined with MMF. This has prompted discussions regarding a possible “paradigm shift” in the treatment of LN, with consideration given to the early use of combination therapies^15,16,17,18^.

Given the relevance of renal involvement in SLE and new approaches to the disease and its treatment, this review article aimed to summarize the main updates in the diagnosis and treatment of LN, based on three recently published international reference guidelines in nephrology and rheumatology, KDIGO (Kidney Disease: Improving Global Outcomes) 2024, EULAR (European League Against Rheumatism) 2023 and ACR (*American College of Rheumatology*) 2025. In addition, it highlights aspects of interest from the national guidelines of the Brazilian Society of Rheumatology (SBR), published in 2024^5,17,18^, with the aim of assisting nephrologists, rheumatologists, and general practitioners in familiarizing themselves with the new recommendations in the field, so as to achieve best practices for the treatment of LN.

## Etiopathogenic Mechanisms

The pathophysiology of the disease is complex and heterogeneous. SLE develops in genetically susceptible individuals, leading to loss of immune tolerance associated with exposure to specific environmental triggers, responsible for stimulating the inflammatory response, such as viral infections, sun exposure, and hormonal changes^19,21,22^. In addition, there is a failure in the neutrophil apoptosis process - NETosis -, which increases the exposure of nuclear antigens to the immune system, contributing to the development of chronic autoimmunity^19,22,23^.

Renal involvement in SLE occurs from the deposition of circulating immune complexes (ICs) in the renal tissue, or from the *in-situ* ICs formation, which may vary in size, affinity, and charge. This explains the presence, in some cases, of mesangial, subendothelial, and subepithelial deposits^24^. Once deposited in renal tissue, these ICs activate the classical complement pathway, the macrophages, and the neutrophils, which also contribute to the amplification of the inflammatory immune response at the site. This process culminates in varying degrees of podocyte injury, mesangial, endothelial, and parietal epithelial cell proliferation, as well as increased synthesis and deposition of extracellular matrix, leading to kidney dysfunction^20–22^.

## Clinical Presentation and Diagnosis

Renal involvement in SLE may remain asymptomatic for a long period; therefore, patients should be actively monitored for potential renal activity. The evaluation should include both clinical and laboratory parameters, involving at least urinary sediment analysis, urine protein-creatinine ratio, and assessment of renal function through serum creatinine measurement and estimated glomerular filtration rate (eGFR) calculation. These assessments should be performed at least at the time of diagnosis and repeated every 6–12 months, even in asymptomatic individuals with SLE^5,25^.

Although changes in urinary sediment and/or kidney function represent important signs of active LN, clinical and laboratory findings do not always correlate with the extent or severity of histological kidney involvement^26,27^. In general, patients with active urinary sediment and proteinuria > 0.5 g/24h - commonly accompanied by microscopic hematuria and/or leukocyturia - particularly if they present with hypocomplementemia and positive anti-double-stranded DNA (anti-ds-DNA), exhibit proliferative classes of LN. It is worth noting that a calculator using the parameters active urinary sediment, serum creatinine, and anti-ds DNA demonstrated good accuracy in predicting proliferative classes (https://nefritelupica.medicalcore.com.br)^28^. However, when available, renal biopsy remains the gold standard for defining the LN class and for differential diagnosis, as well as for grading activity and chronicity, which are tools that support therapeutic decision-making and renal prognosis assessment^5^.

## Kidney Biopsy in Systemic Lupus Erythematosus Patients

In 2003, the International Society of Nephrology and the Renal Pathology Society established the histological classification currently used for LN, which is divided into six classes (according to Table S1), based on light microscopy and immunofluorescence findings. According to these regulations, the glomeruli, tubules, and interstitium should be evaluated, with a description of activity and chronicity indices, as well as the vascular compartment, which may reveal associated conditions such as antiphospholipid antibody syndrome (APS) and other thrombotic microangiopathies, with an estimated prevalence of up to 24%^29^.

The most common and severe type is proliferative glomerulonephritis, which may be focal (class III) or diffuse (class IV). In addition, more than one histological class may be observed during the course of the disease, or even simultaneously^30,31^. The presence of proliferative classes (III and IV) associated with class V is a poor prognostic factor and should be managed aggressively with immunosuppressive regimens^9,30^. In some cases, electron microscopy may also be useful for diagnosis, allowing, for example, the confirmation of lupus podocytopathy - histologically defined by diffuse podocyte process effacement, but without immune deposits along the capillary loops - which distinguishes it from other forms of LN, with an estimated prevalence of up to 2%^31,32^.

In addition to defining the class of LN, kidney biopsy also enables the quantification of activity and chronicity indices, the former being graded from 0 to 24 points and the latter from 0 to 12 points, as described in Table S2. Although no clear definition is provided in the guidelines, patients with predominantly chronic lesions are unlikely to require immunosuppression for LN, but may benefit from antiproteinuric measures, for instance^25^.

In the presence of suspected renal activity, a biopsy should be performed whenever possible, since determining the LN classification based solely on clinical and laboratory parameters is limited. Both EULAR and KDIGO recommend performing a renal biopsy in the presence of proteinuria ≥ 0.5 g/24 hours or in cases of worsening kidney function with no other known cause^5,17^.

The indication for rebiopsy remains controversial, and repeating kidney biopsy in the event of recurrent renal activity is not always necessary^33^. However, emerging data suggest that serial biopsies may assist in ongoing treatment decisions and in predicting renal prognosis. Repeated biopsies demonstrated considerable disagreement between clinical and histological disease activity^25^. Studies have shown that, after 6 to 8 months of immunosuppressive therapy, 20% to 50% of all patients meeting criteria for clinical remission still exhibited histological evidence of active inflammation, while 40% to 60% of patients without histological evidence of disease activity still had persistent high-grade proteinuria^26,34^.

Regarding long-term renal response, evidence of histological activity has been reported in approximately 20% of patients with sustained clinical remission, even after years of immunosuppressive treatment, whereas 40% of patients with persistent residual proteinuria showed complete histological remission^35^. These findings highlight a potential role for rebiopsy as an adjunctive tool in deciding whether to withdraw or continue maintenance therapy (“subsequent” according to the most recently adopted terminology), although no formal guideline recommendations on this practice are currently available^17,25^. The indications for biopsy and rebiopsy are summarized in Table 1.

**Table 1:** Summary of indications for renal biopsy in patients with systemic lupus erythematosus

1st Biopsy – perform whenever possible, when:	
Proteinuria ≥ 0.5 g/24h (with or without other accompanying symptoms) Elevated serum creatinine with no other apparent cause	
**Rebiopsy – possible indications:**	**Observations**
Support in deciding whether to discontinue maintenance therapy (after at least 3 years, including both induction and maintenance phases). Recurrence of renal activity for class reassessment, especially if initial presentation with nonproliferative class. LN refractory to induction therapy, usually after 24 weeks, if other associated diagnoses are suspected or if progression to chronicity occurs. Individualization is required.	Sustained histological activity, even with complete clinical response, suggests continuing maintenance immunosuppression.

## Treatment of Lupus Nephritis

Early diagnosis and timely initiation of treatment are essential in the management of LN, since in its initial stages the disease is usually oligosymptomatic and may progress to chronic kidney disease within a few months if left untreated. As a chronic disease with a high risk of recurrence, immunosuppressive treatment for LN generally includes both an induction phase and a maintenance phase, aimed at preventing further flares and maintaining remission^17^.

### General Aspects of Treatment

**Figure 1:**
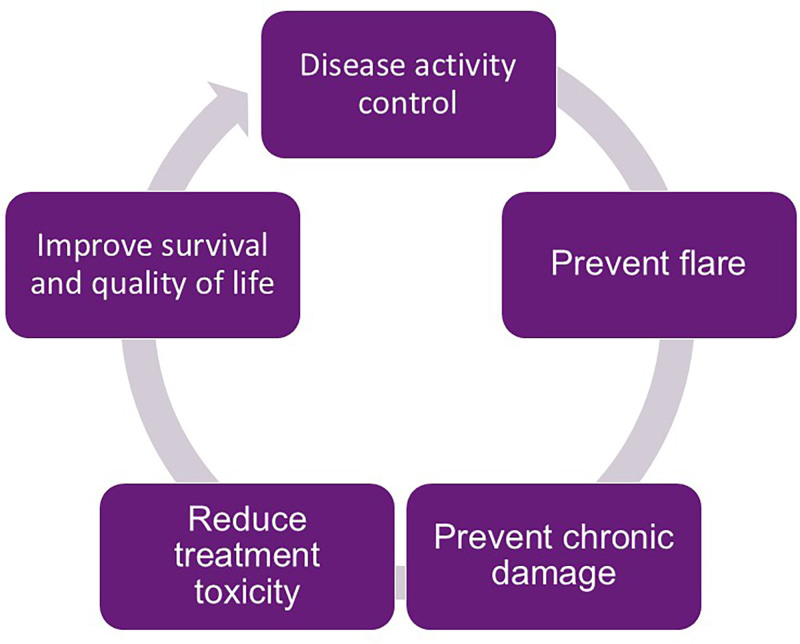
Treatment goals for patients with lupus nephritis. Adapted from Echavarria et al.^84^

In addition to the use of immunosuppressants, several other measures are important, including photoprotection (use of sunscreen with a SPF ≥ 30, preferably > 50, and limiting the area of exposure), adjuvant measures to reduce progression to chronic kidney disease, control of cardiovascular risk factors, prevention of infections, family planning and contraception, as well as bone health assessment^5,17^. Below, we will address some of the main general treatment measures, noting that our treatment goals should go far beyond the mere induction of remission (Figure 1).

#### Cardiovascular Risk

Cardiovascular complications are an important cause of morbidity and mortality in individuals with SLE, due to both traditional and non-traditional risk factors. Furthermore, the use of corticosteroids and calcineurin inhibitors may lead to adverse events such as systemic arterial hypertension (SAH), dysglycemia, dyslipidemia, and other aspects related to increased cardiovascular risk. Thus, in addition to controlling disease activity, regular assessment of traditional cardiovascular risk factors and the implementation of measures to reduce them, such as lifestyle changes (smoking cessation, weight control, and regular physical activity), as well as control and treatment of blood glucose, SAH, and dyslipidemia, are crucial for preventing cardiovascular events^5,17^.

#### Risk of Infection

Currently, this represents one of the leading causes of death in this population, particularly during the initial phase of treatment, when immunosuppression is most intense^36,37^. Therefore, preventive strategies should be implemented to mitigate this risk. The use of sulfamethoxazole/trimethoprim is not formally recommended by the guidelines reviewed, in contrast to other diseases, such as ANCA-associated vasculitis, mainly due to the occurrence of sulfonamide-related adverse reactions in a considerable number of cases. However, some studies suggest prophylaxis in patients receiving immunosuppressants (CYC or MMF), prednisone at doses ≥ 20 mg for a period longer than 4 weeks, active nephritis, and/or interstitial lung disease^38^.

Updating the vaccination schedule according to age group and immunosuppressive status should always be encouraged, preferably when the disease is inactive and prior to the initiation of any immunosuppressive therapy. Inactivated vaccines, as well as subunit, peptide or protein-based, and genetic vaccines are safe at any stage of treatment, while live-attenuated vaccines should be avoided during immunosuppression^5^. It is recommended that immunizations against infections, including influenza, pneumococcal, meningococcal, COVID-19, *Herpes zoster* (inactivated recombinant vaccine), and HPV be administered^5,17^.

#### Chronic Kidney Disease (CKD)

As with any other cause of CKD, measures to slow its progression are of paramount importance in LN, including adequate blood pressure control, avoidance of a high-protein and sodium-rich diet, smoking cessation, and maintenance of regular physical exercise. Regarding specific pharmacological measures, renin-angiotensin system blockade (angiotensin-converting enzyme inhibitors [ACEIs] or angiotensin receptor blockers [ARBs]) is recommended for patients with SAH or with proteinuria > 300 mg/24h (suggested if > 30 mg/24h). Sodium-glucose co-transporter 2 inhibitors (SGLT2i) may be considered in combination with ACE inhibitors or ARBs if eGFR > 20 mL/min/1.73m^2^, especially in the presence of albuminuria > 0.2 g/g (urinary creatinine). However, prospective studies demonstrating the benefit of this drug specifically in the LN population are still lacking^39^.

#### Bone Health

Glucocorticoid therapy, particularly at doses ≥ 5 mg/day for more than three months, accelerates bone mass loss. Therefore, calcium supplementation (optimal intake of 1,000 to 1,200 mg/day), when required, and vitamin D supplementation, aiming to maintain serum levels between 30 and 60 ng/mL, are recommended for patients receiving corticosteroids. The use of antiresorptive or anabolic drugs should be considered according to individual assessment of fracture risk, which can be estimated based on demographic data, the patient’s medical history, FRAX (Fracture Risk Assessment Tool) score, and glucocorticoid-induced osteoporosis^5^.

#### Contraception

Pregnancy in individuals with LN is associated with worse maternal-fetal outcomes compared to the healthy population. In addition, some medications used in the treatment of SLE, such as MMF, CYC, methotrexate, and leflunomide, are contraindicated during pregnancy. Therefore, all women of childbearing age should receive counseling on pregnancy and contraception in order to identify potential contraindications and determine the most appropriate time for conception^5^. Currently, it is recommended that pregnancy be planned for a period of at least 6 months of disease control, without the use of any contraindicated drugs during pregnancy. Contraceptive methods containing estrogen should be avoided in patients with active disease, a prior history of cardiovascular events, or an increased risk of thromboembolic phenomena, such as in cases of APS^40,41^.

#### Hydroxychloroquine

Both the updated KDIGO and EULAR guidelines recommend that all patients with SLE receive treatment with hydroxychloroquine (HCQ), with a grade 1 recommendation, unless contraindicated^5,17^. HCQ provides multiple benefits for individuals with SLE through both direct and indirect effects, including: improved disease activity and reduced relapses; improved skin lesions and joint complaints; reduced cumulative doses of corticosteroids; enhanced effect of other medications, such as MMF; prevention of damage; improvement in lipid profile and glucose levels; reduction in the risk of metabolic syndrome and thrombotic phenomena; and a decrease in the mortality risk. This class of drugs has been used in the treatment of SLE for over 50 years and, among antimalarial agents, HCQ should be preferred over chloroquine diphosphate due to its better safety profile^17,42^.

Antimalarial drugs are melanotropic and concentrate in ocular structures containing melanin, such as the retinal pigment epithelium and the choroid. Their accumulation in the retina may affect photoreceptor cells, particularly the cones in the macula, the central part of the retina responsible for vision. The risk of retinal toxicity is higher with chloroquine diphosphate than with HCQ. It is important to emphasize that ocular lesions and the establishment of damage may be asymptomatic, and symptoms, when present, may emerge late. The main symptoms include decreased visual acuity, changes in color perception, spots or areas of vision loss (scotomas), and blurred vision^43^.

Currently, the recommended dose of HCQ is 5 mg/kg/day, with a maximum dose of 400 mg/day. For patients with eGFR < 30 mL/min/1.73m^2^, the dose should be reduced by 30–50%^5,44,45,46^. At the recommended doses, the risk of toxicity is less than 1% within 5 years of use and less than 2% within 10 years; however, it increases to nearly 20% after 20 years of treatment. After this period, even patients with no prior toxicity have a 4% risk in the subsequent year. The main risk factors for ocular toxicity are daily doses above the recommended level, duration of use, kidney failure, pre-existing maculopathy or retinopathy, and concomitant use of tamoxifen. Other risk factors include advanced age, liver failure, and genetic factors possibly related to abnormalities in the ABCA4 gene or cytochrome P450^47^.

In 2016, the American Academy of Ophthalmology updated its recommendations for screening retinopathy in patients receiving chloroquine diphosphate or HCQ. It is recommended that patients starting the drug undergo an eye exam within the first year of treatment, including a fundus examination. Visual field testing and spectral domain-optical coherence tomography (SD-OCT), although useful at treatment initiation, are not mandatory unless the patient has risk factors or other conditions (e.g., pre-existing maculopathy, glaucoma, or others) that may affect baseline screening tests. In the absence of major risk factors, screening tests may be performed annually starting five years after the baseline evaluation. If risk factors are present, screening tests should be conducted annually, with automated visual field testing and SD-OCT being recommended. In certain situations, additional tests may be indicated, such as multifocal electroretinography (mfERG), which provides objective information on the visual field, and fundus autofluorescence (FAF), which can topographically reveal damage, especially in Asian patients^47^. SD-OCT and visual field testing are more sensitive techniques, with potential for early detection of HCQ-related changes. It is worth noting that the typical “bull’s eye maculopathy” pattern should not be expected for the diagnosis of antimalarial drug toxicity, as this finding is late and irreversible^48^.

### Treatment of Class i and ii Lupus Nephritis and Lupus Podocytopathy

Patients with mesangial classes of LN (I and II) generally have a benign course, with microscopic hematuria and low-grade proteinuria. In these cases, the initiation of specific immunosuppression is not necessary unless there are other target-organ lesions that require immunosuppressive treatment^49^.

According to the KDIGO guidelines for lupus nephritis, in patients with a class I or II histological pattern on light microscopy but clinically manifesting nephrotic syndrome, lupus podocytopathy should be considered. The diagnosis may be confirmed by electron microscopy, which shows diffuse podocyte foot process effacement and no subepithelial or subendothelial deposits. In such cases, the disease usually behaves similarly to primary podocytopathies (minimal change disease/focal segmental glomerulosclerosis) and should be managed accordingly, using glucocorticoid therapy, which generally yields good results^32,50,51^. However, in lupus podocytopathy, there appears to be a greater tendency toward recurrence, although data remain scarce. Maintenance therapy should be considered in this context, although the optimal duration and agent are not well defined. Options include low-dose glucocorticoids in combination with MMF, azathioprine, or CNI^5,52^. EULAR does not provide specific recommendations for classes I and II.

### Treatment of Class iii And iv Lupus Nephritis

Proliferative classes are the most frequently diagnosed, presenting the highest likelihood of progression to loss of kidney function, with a prevalence of stage V CKD of up to 30% at 10 years^53^. In recent decades, the treatment of these classes has undergone major changes: from the isolated use of corticosteroids until the 1970s, to the use of CYC associated with corticosteroids up to the 2000s, resulting in a significant impact on both kidney and overall survival in individuals with SLE. In the early 2000s, the first studies on MMF were published, establishing it as an effective alternative with the potential for fewer adverse effects. Thus, in recent decades, first-line induction therapy for LN has been restricted to the use of MMF (2–3 g/day of mycophenolate mofetil) or intravenous CYC, either at a dose of 500 mg every 2 weeks for 3 months (Euro-Lupus Nephritis Trial protocol) or in monthly doses of 0.5–1.0 g/m^2^ body surface area for 6 months (National Institutes of Health [NIH] protocol, USA)^54,55,56^. It should be noted that, in the present text, the term “mycophenolate (MMF)” may refer to either mycophenolate mofetil or mycophenolate sodium, with the reported doses most often referring to the former, although both are used in clinical practice.

Interestingly, after nearly 20 years without the approval of new therapies, several randomized trials have emerged in recent years demonstrating new treatment options^5^. In 2020, the BLISS-LN study was published, evaluating belimumab - a monoclonal antibody that inhibits BAFF (B-cell activating factor) - associated or not with standard therapy (CYC Euro-Lupus or MMF) in classes III or IV(±V) or active class V LN. The study included 448 randomized patients who were followed for 2 years^57^. A higher proportion of patients achieved complete remission, as well as an effective primary renal response, particularly among those with a baseline urinary protein-creatinine ratio below 3 g/g. Furthermore, the addition of belimumab was associated with greater preservation of renal function and fewer relapses^57,58,59^.

In 2021, the AURORA 1 trial, which evaluated voclosporin (a more potent calcineurin inhibitor, with a lower incidence of adverse events than cyclosporine and tacrolimus, and without the need for serum level monitoring) in combination with low-dose MMF and prednisone, demonstrated a higher likelihood of achieving complete remission compared with MMF and prednisone alone^60^. In 2015, a study in an Eastern population receiving multitarget treatment (mycophenolate mofetil 1 g/day and tacrolimus 4 mg/day) had already demonstrated the benefit of combining calcineurin inhibitors in the induction treatment of patients with proliferative LN; however, due to the ethnic restriction of the study, these results were not sufficient to justify the inclusion of this class of drugs as first-line treatment^61^. More recently, the AURORA 2 trial, with a 3-year follow-up, demonstrated a sustained higher remission rate without worsening of renal function in patients^62^.

Given the higher remission rates, with no increased risk of adverse events, and the limited therapeutic options with a good safety profile available, both belimumab and voclosporin have been approved in some countries as treatment options for class III or IV (±V) LN, always in combination with standard therapy. Based on all this new evidence, both the KDIGO guidelines and the European, American and Brazilian rheumatology guidelines have issued updates with new recommendations for the treatment of classes III and IV (±V) LN.

EULAR and KDIGO describe four first-line regimens for the treatment of proliferative classes, associated with prednisone and hydroxychloroquine, as listed below (Table 2)^5,17^.

**Table 2:** First-line regimens for the treatment of proliferative classes, according to eular and kdigo

Options for first-line induction therapy in proliferative classes	Observations
Cyclophosphamide monotherapy	Preference for a reduced dose of 500 mg every 14 days for 3 months, corresponding to the Euro-Lupus regimen
Mofetil mycophenolate monotherapy	Dose of 2-3 g/day
Belimumab in combination	Dose of 10 mg/kg IV on D0, D14, D28, and every 28 days thereafter With mycophenolate or cyclophosphamide Not indicated if eGFR < 30 ml/min/1.73m^2^
Voclosporin associated with mycophenolate mofetil 2 g/day	Alternatives: mycophenolate mofetil 1 g + tacrolimus (0.05–0.1 mg/day, divided into 2 doses, adjusted according to serum levels) or cyclosporine (3 mg/kg/day, divided into 2 doses, adjusted according to serum levels), in the absence of voclosporin. Not indicated if eGFR < 45 ml/min/1.73m^2^

There are several similarities between both international guidelines. Neither establishes a clear hierarchy among the regimens, requiring an assessment of the risk of poor adherence, the profile of adverse effects, and extrarenal manifestations in the decision-making process. In patients with nephrotic range proteinuria, calcineurin inhibitors are preferred, while belimumab therapy has shown lower efficacy in this population. Both consider the importance of proteinuria reduction and maintenance/improvement of renal function as early parameters for assessing renal response, considering a > 25% decrease in proteinuria within 3 months as a sign of good clinical response, associated with stable renal function, with a maximum eGFR reduction of 20% from baseline. In addition, they emphasize the importance of using immunosuppressants for at least 3 years, with their withdrawal preferably performed in a slow and progressive manner.

Regarding the corticosteroid use, there is consensus that they should be the first immunosuppressive drugs to be discontinued, with caution and a slow, gradual taper, due to the risk of recurrence^63^. Currently, there is also a trend toward the use of reduced doses of corticosteroids in induction therapy. Pulse therapy with methylprednisolone, at doses of 125 to 500 mg for 1 to 3 days, is recommended, in addition to the introduction of lower doses and a more rapid tapering of oral corticosteroids, aiming to achieve doses ≤ 5 mg/day in 3 to 6 months. This tendency is particularly applicable to less severe patients who are progressing favorably, since reduced doses show a similar response with a lower risk of adverse events^60^.

Regarding several other issues, there is no agreement between both guidelines. In more severe cases, KDIGO does not consider any change in treatment necessary, merely stating that the combination with belimumab may be seen as an option for patients at higher risk of progressing to CKD, such as those already presenting with impaired kidney function or high activity and/or chronicity indices. EULAR proposes that high-dose CYC be reserved, according to the NIH protocol, for patients at high risk of kidney dysfunction, especially those who already have severe kidney dysfunction and/or show evidence of cellular crescents, fibrinoid necrosis, and/or severe interstitial inflammation on biopsy. The Euro-Lupus protocol is considered a preferable option for other cases due to its lower cumulative dose and reduced toxicity. KDIGO, on the other hand, presents both intravenous protocols as initial options, emphasizing that the Euro-Lupus protocol was tested in a predominantly Caucasian population, whereas the NIH study involved a multiethnic population. KDIGO also mentions oral CYC as an alternative, with the same efficacy, although it entails a regimen with a higher cumulative CYC dose and greater toxicity.

Despite the absence of a hierarchy among treatments in KDIGO, consideration should be given to favoring regimens that include belimumab in patients with a history of relapses, while calcineurin inhibitors should be prioritized in cases of intolerance to high doses of MMF or in the presence of proteinuria > 3 g/24 h, as previously mentioned. Multitarget regimens may also be considered if there is difficulty in tapering prednisone doses.

As for maintenance treatment, KDIGO recommends MMF as the first choice for most patients, with the main exception being those planning to conceive, in which case azathioprine is preferred. In contrast, EULAR considers that maintenance treatment should be decided according to induction: patients initially treated with CYC alone could use either azathioprine or MMF and those who received induction with MMF would continue with the same drug. Both guidelines consider that those who underwent triple therapy should maintain this treatment for a period of 3 years.

### Treatment of Class v Lupus Nephritis

The incidence of isolated class V LN ranges from 5-20%. This form may be associated with proliferative classes and is related to a higher risk of nephrotic syndrome and renal vein thrombosis^5,64,65,66^. The risk of loss of kidney function is associated with the severity of proteinuria and, unlike what is described in primary membranous glomerulopathy, does not usually present with spontaneous remission^65,67,68^. Thus, class V LN is a potentially severe form, and the decision regarding treatment should be based primarily on the degree of proteinuria, but also on the presence of progressive loss of kidney function.

Considering proteinuria levels as a criterion for treatment indication, there is a discrepancy between the two guidelines. EULAR recommends the use of immunosuppressants in cases of persistent proteinuria > 1 g/24 h, whereas KDIGO advises initiating immunosuppression especially in patients with nephrotic syndrome^5,17^. In this case, the lack of consensus is mainly explained by smaller samples of pure class V in randomized studies. For this reason, we suggest individualizing treatment in patients with proteinuria > 1 g/24 h, considering the stability and severity of proteinuria, renal function, in addition to the risks and benefits of treatment and disease activity in other organs^5^.

Regarding the immunosuppressive regimen to be used, there is no high-quality evidence to guide the recommendation for class V LN without an associated proliferative component. A small randomized clinical trial demonstrated higher remission rates with the combination of CYC and corticosteroids, as well as with CNI and corticosteroids, when compared to the use of corticosteroids alone. However, a higher recurrence rate was observed in patients receiving CNI compared to CYC^69^. In addition to CYC and CNI, other small studies have also evaluated the efficacy of MMF, azathioprine, and rituximab (RTX) in combination with corticosteroids, with response rates ranging from 40% to 60%^5^.

In the AURORA study, in which 14% of patients had isolated class V LN, the addition of voclosporin to standard therapy (MMF and corticosteroids) proved more effective than standard immunosuppression alone in achieving renal response in 31 patients with class V LN. When analyzing another outcome, although not statistically significant, a shorter mean time to proteinuria reduction to 0.5 mg/mg was observed in patients treated with voclosporin in combination with corticosteroids and MMF (3.6 months) compared with controls treated with corticosteroids, MMF, and placebo (8.3 months)^60^. With regard to belimumab, results from a post-hoc analysis of the BLISS-LN study (16% with pure class V LN) suggested that patients with nephrotic range proteinuria did not experience a benefit on the primary outcome, although a trend toward reductions in other outcomes, such as >30% eGFR decline and a reduction in the number of relapses, was observed^59^.

Despite the lack of recommendations based on robust evidence, KDIGO 2024 supports the use of MMF, CNIs, azathioprine, RTX, or short-term use of CYC^5^. EULAR, in turn, recommends MMF as the first choice, with CNIs and CYC as alternative options and RTX reserved for patients with refractory class V LN^70^. In 2023, the Spanish Society of Nephrology published a guideline that further individualized the approach, suggesting a regimen with CNIs and MMF for patients with nephrotic syndrome. For patients without nephrotic syndrome but with proteinuria > 1 g/24h, the guideline recommends administration of CNIs^71^.

### Response Criteria and Therapeutic Target

The assessment of response to therapy is based on improvement in proteinuria and stabilization or improvement in kidney function. However, there is no universally accepted criterion regarding the level of improvement required, which hinders direct comparison across different clinical trials^5^. Post-hoc analyses of two large European randomized trials indicated that proteinuria levels after 12 months of treatment are the best predictor of long-term renal outcomes, with improved outcomes (risk of end-stage kidney disease or doubling of serum creatinine within 10 years) when proteinuria levels < 0.7-0.8 g/24h were achieved^72,73,74,75,76^. EULAR therefore reinforces that therapy should aim for a proteinuria target of <0.5–0.7 g/24h within 12 months, although up to 50% of patients who do not achieve this target may also maintain stable kidney function in the long term^70,73,77^.

The ALMS study analysis suggested that a 25% reduction in proteinuria levels and normalization of complement levels within 8 weeks of treatment predict favorable renal outcomes. This finding provided the basis for EULAR to consider a >25% reduction in proteinuria as a treatment target (in this case, within 3 months), together with improvement/stabilization of renal function, and a >50% reduction in proteinuria at 6 months^70,78,79^. The proteinuria targets for assessing response to therapy during the first year of induction treatment are summarized in Table 3.

**Table 3:** Factors to be considered when choosing immunosuppressive therapy in clinical practice

	MMF	CYC NIH	CYC Euro-Lupus	AZA	TAC	CsA	BEL	RTX
Promotes adherence		X	X					
Ease of access	X	X	X	X		X		
Need for infusion center		X	X				X	X
Severity or refractoriness		X					X	X
Pregnancy/lactation				X	X	X		
Risk of infertility		X	X					
High cost					X		X	X

Abbreviations – TAC: Tacrolimus; CsA: Cyclosporine A; MMF: Mycophenolate Mofetil; AZA: Azathioprine; CYC: Cyclophosphamide; BEL: Belimumab; RTX: Rituximab. Note – Adapted from SBR table.

Still on the subject of treatment response (Table 4), KDIGO emphasizes that improvements in proteinuria and eGFR occur continuously over time, with the rate of improvement varying considerably among patients. Furthermore, there are notable differences between proteinuria and baseline eGFR at the time of disease presentation, which also influence the response time. Nephrotic-range proteinuria, for example, may require more than 6–12 months to normalize or decrease^5,80^. Although this guideline also emphasizes the favorable prognosis associated with a > 25% reduction in proteinuria at 3 months and < 0.7-0.8 g/24 h at 12 months, particularly in cases of nephrotic range proteinuria, it is not strictly necessary for these time frames to be achieved for decision-making purposes. According to KDIGO, it is acceptable to wait longer (18 to 24 months) to obtain a complete response in patients showing continuous improvement^5^.

**Table 4:** Criteria for defining types of response to treatment in lupus nephritis, according to the 2024 kdigo guidelines

Types of responses	Definition
Complete renal response	Reduction of proteinuria to <0.5 g/g P/C or 24-hour proteinuria Stabilization or improvement of renal function (value within ±10%–15% of baseline eGFR) Within 6 to 12 months after initiation of therapy
Primary efficacy renal response	P/C < 0.7 g/g Maximum reduction in eGFR of 20% from baseline or eGFR > 60 ml/min/1.73 m^2^ No use of rescue therapy Associated with better renal outcomes
Partial renal response	Reduction in proteinuria by at least 50% and to <3 g/g P/C or 24-hour proteinuria Stabilization or improvement in renal function (±10%–15% of baseline) Within 6 to 12 months after initiation of therapy
No response	No CR or PR criteria after 6–12 months of ISS

Abbreviations – ISS: Immunosuppression; P/C: protein-creatinine ratio in urine; CR: Complete Remission; PR: Partial Remission; eGFR: Estimated Glomerular Filtration Rate. Note – Adapted from KDIGO table.

### Refractory Lupus Nephritis

Assessment of response is traditionally performed at 8 to 12 weeks, but if the patient shows no reduction in proteinuria and/or improvement in kidney function at 4 weeks, the prognosis is considered unfavorable, and investigation of possible causes of refractoriness should be addressed.

The first step recommended by KDIGO in refractory cases is to assess treatment adherence, considering, in such cases, the use of CYC (given its intravenous administration). Some studies assessing serum levels, especially with hydroxychloroquine, have shown a direct correlation between serum levels and higher incidence of recurrence, highlighting the importance of both adherence assessment and inter-individual dose variability^81^.

If adherence is confirmed, a new renal biopsy is recommended to assess the possibility of findings that may justify the lack of response, such as the presence of high chronicity indices and thrombotic microangiopathy. If reversible renal activity is confirmed while on optimized treatment, the recommendation is to switch to a different first-line regimen from the initial one.

Finally, in the absence of response to available regimens, second-line treatments such as RTX, prolonged high-dose CYC, as well as enrollment in randomized trials with novel drugs should be considered^5^.

It should be emphasized that, in addition to the medications currently recommended by the KDIGO and EULAR guidelines, a new option has shown promise in the induction treatment of lupus nephritis: obinutuzumab. It is a humanized anti-CD20 monoclonal antibody, whose efficacy was recently evaluated in the REGENCY study, a phase 3 clinical trial, in combination with standard therapy with mycophenolate mofetil. The primary outcome assessed was the complete renal response rate at week 76, defined as a urine protein-creatinine ratio < 0.5 and eGFR ≥ 85% of baseline. As a result, 46.4% of patients in the obinutuzumab group achieved complete renal response, compared with 33.1% in the placebo group. Despite the favorable findings, further studies are needed to support the formal incorporation of obinutuzumab as a therapeutic option for patients with active lupus nephritis^82^.

## Highlights from the Brazilian Society of Rheumatology Consensus for the Diagnosis and Treatment of Lupus Nephritis

In addition to the main points raised by international guidelines, it is also worth mentioning the main recommendations regarding the management of LN in the Brazilian context. In this regard, in 2024, the SBR published updates to the treatment recommendations for LN, with 14 recommendations (Table S3) developed according to the GRADE methodology, considering the drug access and availability at the national level. The document also presents a proposed treatment flowchart and suggestions for clinical practice. Moreover, several factors inherent to the patient should also be considered when choosing therapy. Among the factors that may assist in decision-making regarding immunosuppressive therapy (Table 3), the following stand out: patient adherence to medication, availability, access, need for an infusion center, severity or refractoriness, prior relapses, compatibility with pregnancy/lactation, risk of infertility, and medicines costs.

**Figure 2.**
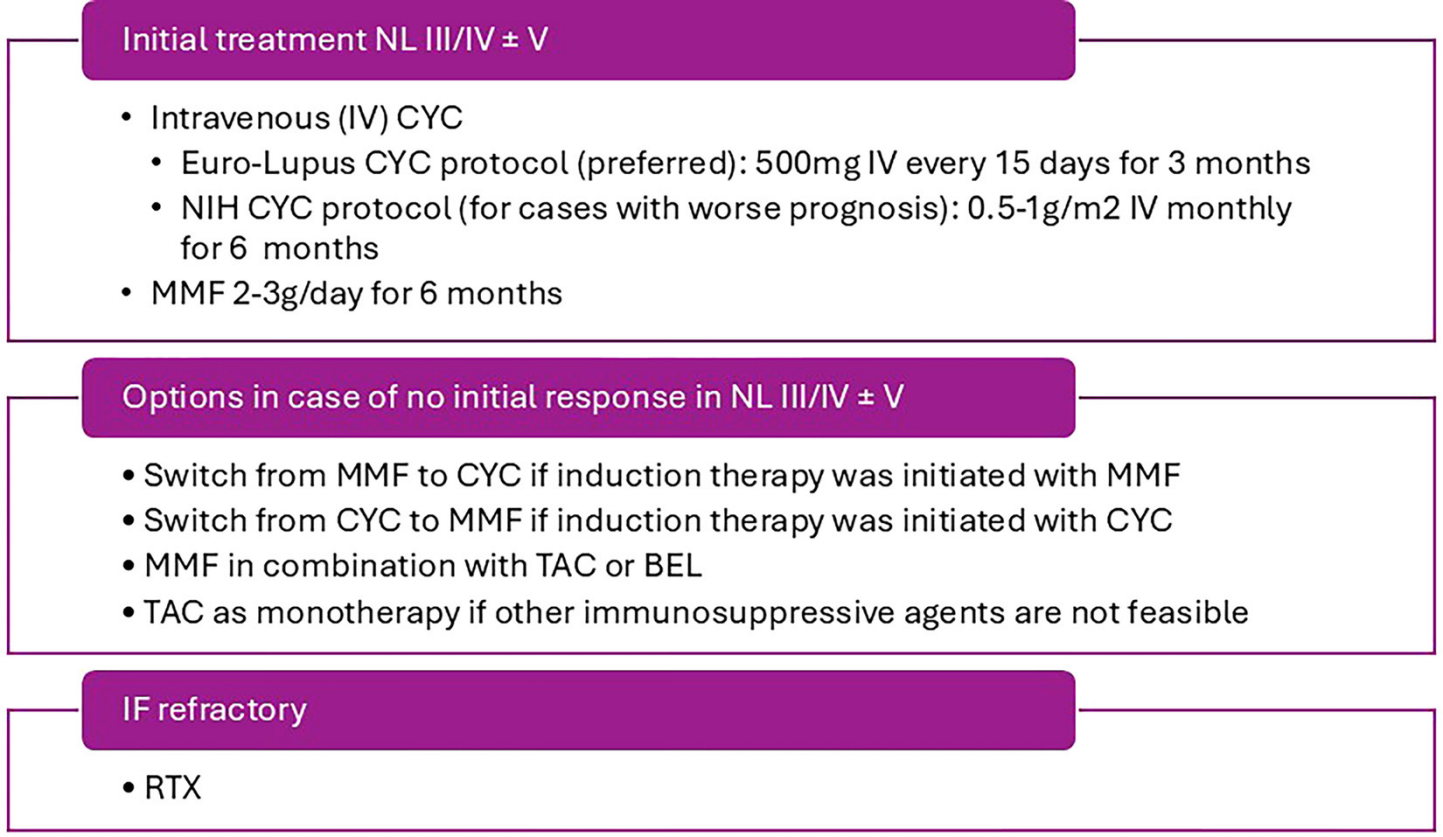
Induction therapy for proliferative classes of LN, according to the SBR. Adapted from Reis-Neto et al.^18^

The SBR flowchart suggests initiating induction treatment for proliferative classes with MMF monotherapy or intravenous CYC. In agreement with EULAR, the SBR flowchart also reinforces the need to achieve the target renal response, which should be frequently reassessed within the first year of treatment (at 3 months, 6 months, and at the end of 1 year, as described in Table 5). In the absence of response to the initial regimen, switching regimens or using combination therapy is recommended, as described in Figure 2. Tacrolimus monotherapy is also an option for cases in which other drugs cannot be used. In the event of therapeutic failure with at least two induction therapies or contraindications to standard treatments, after reviewing factors such as drug adherence, immunosuppressive drug dosage, and concomitant comorbidities, as well as considering the need for rebiopsy, the use of RTX is indicated. The initial induction treatment suggested for class V LN is also described in Table S4. When choosing medication, the severity of the condition should be considered, according to proteinuria and serum albumin levels, in addition to the individual patient factors mentioned above.

**Table 5:** Proteinuria targets in the first year of treatment

Target renal response – proteinuria
Reduction ≥ 25% in 3 months
Reduction ≥ 50% in 6 months
Reduction to < 0.7–0.8 g/24h in 12 months

Abbreviations – TAC: Tacrolimus; MMF: Mycophenolate Mofetil; AZA: Azathioprine; CYC: Cyclophosphamide; BEL: Belimumab; RTX: Rituximab; NIH: National Institutes of Health protocol.

Since universal access to renal biopsy is not a possibility in Brazil, the SBR consensus emphasizes that when biopsy is not available or there are contraindications to the procedure, the therapeutic decision may be based on clinical and laboratory parameters, since delays in initiating immunosuppressive treatment (especially in suspected cases of rapidly progressive glomerulonephritis) are associated with poorer short- and long-term renal prognosis^25^.

The SBR Consensus on LN further highlights the importance of adjuvant measures to immunosuppressive therapy for a good response to treatment, including photoprotection, patient information and education, blood pressure control, use of antiproteinuric medications, cardiovascular risk assessment and proteinuria control, treatment of corticosteroid-induced osteoporosis, investigation of antiphospholipid antibodies and prevention of thromboembolism, immunization and prevention of infections, as well as the avoidance of smoking and nephrotoxic drugs.

## Highlights from the American College of Rheumatology Consensus on the Diagnosis and Treatment of Lupus Nephritis

**Figure 3.**
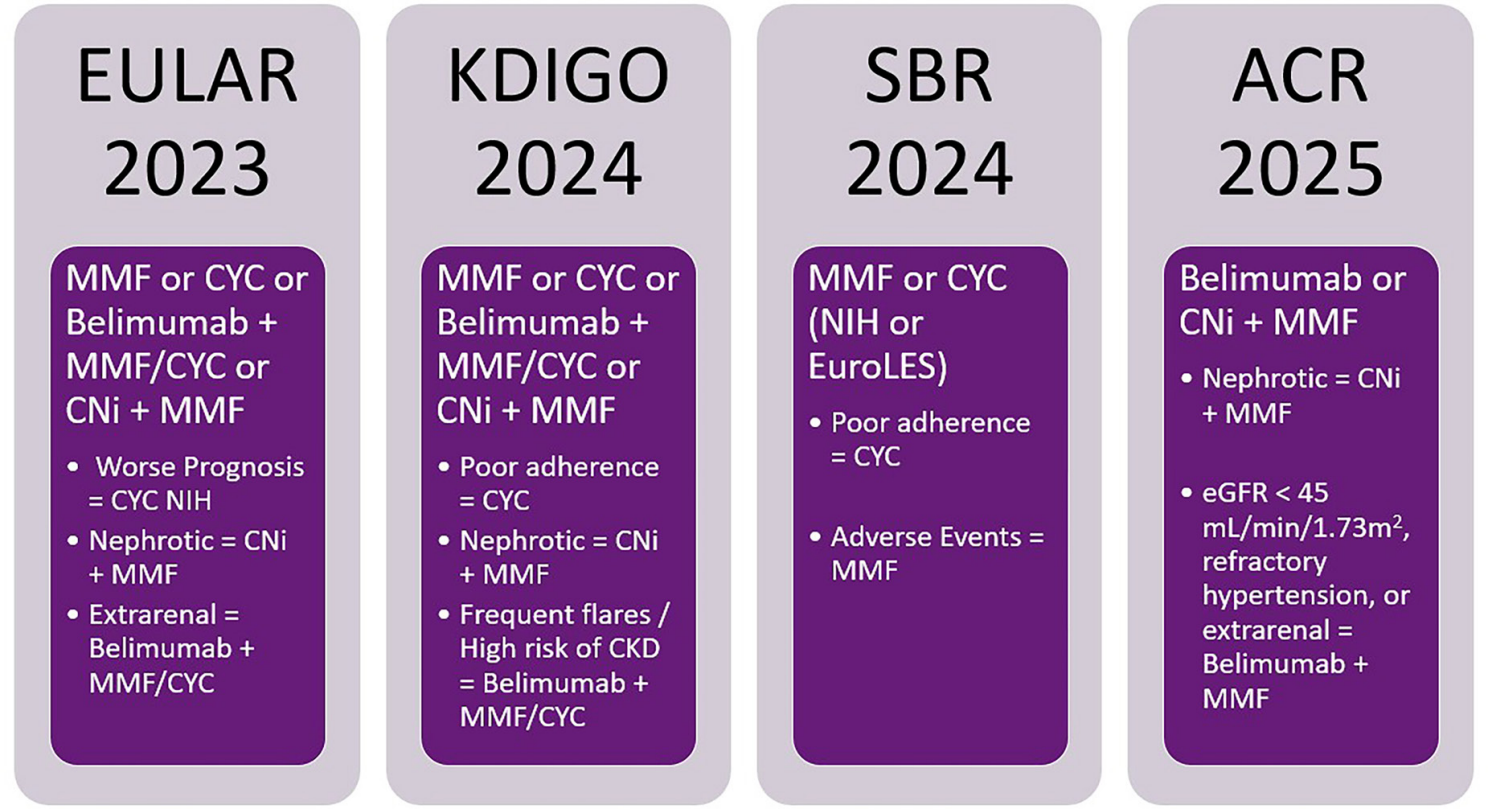
Comparison of first-line treatment guidelines for proliferative lupus nephritis.

Complementing the analysis of the main guidelines currently in force on the management of lupus nephritis, it is worth highlighting relevant aspects of the ACR Consensus, published in May 2025. This document presents some therapeutic recommendations that differ from those previously established by other societies and described herein, in relation to LN induction therapy, as summarized in Figure 3 ^83^.

Abbreviations – MMF: Mycophenolate Mofetil; CYC: Cyclophosphamide; CNI: Calcineurin Inhibitor; NIH: National Institutes of Health protocol; CKD: Chronic Kidney Disease; eGFR: Estimated Glomerular Filtration Rate.

The ACR recommends only triple therapy regimens as first-line treatment for proliferative LN: belimumab or a calcineurin inhibitor combined with mycophenolate and prednisone, considering that recent results from the AURORA and BLISS-LN 2 studies have demonstrated the superiority of these regimens, thereby justifying the hierarchical difference between triple therapies compared to dual therapies^59,60,62^. In the decision-making process between belimumab and CNIs, certain issues were considered by the consensus: 1) belimumab should be prioritized in patients with eGFR < 45 ml/min/1.73m^2^, with a high chronicity index or blood pressure > 165 × 105 mmHg, due to nephrotoxicity and uncontrolled blood pressure associated with CNIs; 2) patients with extrarenal manifestations should be considered a priority for the use of belimumab, due to its beneficial effect, particularly on skin and joint involvement; 3) patients with proteinuria ≥ 3g/g (if eGFR > 45 ml/min/1.73m^2^) should receive CNI, as belimumab has not demonstrated a benefit over dual regimens in inducing remission in these patients^59,83^.

Another highlight of the consensus was the decision to prioritize the use of MMF over CYC, considering the lower risk of malignancy and infertility associated with the former. However, cyclophosphamide is still considered a good option for patients with gastric intolerance to mycophenolate, a history of poor adherence, or those presenting with rapidly progressive glomerulonephritis and/or fibrinoid necrosis. The consensus also emphasizes the importance of minimizing corticosteroid use, establishing a maximum dose of 0.5 mg/kg/day or 40 mg/day after pulse therapy.

As for the other classes, emphasis should be placed on the recommendations for class V, in which proteinuria is also considered a determining factor for treatment selection. Triple therapy with CNI, MMF, and corticosteroids is recommended as first-line treatment in patients with proteinuria > 1 g/24 h, while MMF, corticosteroids, azathioprine, or CNI alone are the suggested options for patients with proteinuria < 1 g/24 h.

## Conclusions

The management of LN is one of the areas in nephrology that has undergone major changes in recent years, particularly with regard to induction therapy options, which explains why rheumatology and nephrology societies have issued new treatment recommendations in recent months. Although these guidelines are based on the same studies, they present some differences in perspective. Understanding their similarities and differences could assist in decision-making for the individualized treatment of our patients.

## Data Availability

The entire data set supporting the text of this review article is available and can be accessed from the bibliographic references cited.

## Supplementary Material

The following online material is available for this article:

Table S1 - 2003 ISN/RPS histological classification for lupus nephritis.

Table S2 - Criteria comprising activity and chronicity indices in the assessment of proliferative lupus nephritis.

Table S3 - SBR principles and recommendations for the treatment of proliferative classes of LN.

Table S4 - Induction therapy for class V LN according to SBR.
