# Supplementary material for: Current treatment of lupus nephritis: an overview of the new guidelines
Source: J Bras Nefrol. 2025 Oct 13;47(4):e20250092. doi: 10.1590/2175-8239-JBN-2025-0092en (PMC12520630; doi:10.1590/2175-8239-JBN-2025-0092en)
Supplement: Table S1 - [file 2175-8239-jbn-47-4-e20250092-suppl1.pdf]

*Braz. J. Nephrol.*
https://doi.org/10.1590/2175-8239-JBN-2025-0092en

# Supplementary Material to "Current treatment of lupus nephritis: an overview of the new guidelines"

**Table S1 –** Histopathological classification by ISN/RPS 2003 for lupus nephritis.

| Class | Histological findings |
|---|---|
| I – Minimal mesangial | Immunofluorescence with mesangial deposits and normal light microscopy |
| II – Mesangial proliferative | Mesangial proliferation without endocapillary or subepithelial lesions |
| III – Focal proliferative | Endocapillary proliferation in < 50% of glomeruli<br><br>Classified as: active, active/chronic and chronic (A, A/C e C) |
| IV – Diffuse proliferative | Endocapillary proliferation in > 50% of glomeruli<br><br>Classified as: active, active/chronic and chronic (A, A/C e C) and as segmental (if > 50% of glomeruli have segmental lesions) or global (if > 50% of glomeruli have global lesions) – S or G |
| V – Membranous | Presence of subepithelial deposits detected by light, electronic, or immunofluorescence microscopy. It may be associated with other classes. |
| VI – Advanced sclerosis | Global glomerulosclerosis in > 90% of glomeruli and no active lesions |

Abbreviations – ISN/RPS: International Society of Nephrology / Renal Pathology Society
