# Supplementary material for: Current treatment of lupus nephritis: an overview of the new guidelines
Source: J Bras Nefrol. 2025 Oct 13;47(4):e20250092. doi: 10.1590/2175-8239-JBN-2025-0092en (PMC12520630; doi:10.1590/2175-8239-JBN-2025-0092en)
Supplement: Table S2 - [file 2175-8239-jbn-47-4-e20250092-suppl2.pdf]

Braz. J. Nephrol.
https://doi.org/10.1590/2175-8239-JBN-2025-0092en

Supplementary Material to "Current treatment of lupus nephritis: an overview of the new guidelines"

Table S2 – Criteria that compose activity and chronicity indices in the assessment of proliferative lupus nephritis.

**Activity criteria**	**Scores**	**Chronicity criteria**	**Scores**
Endocapillary proliferation	0-3	Global glomerulosclerosis	0-3
Interstitial inflammation	0-3	Fibrous Crescents	0-3
Hyaline deposits	0-3	Tubular atrophy	0-3
Fibrinoid necrosis (x2)	0-3	Interstitial fibrosis	0-3
Cellular/fibrocellular crescents (x2)	0-3		
Neutrophils/karyorrhexis	0-3		
**Total**	**0-24**	**Total**	**0-12**

Note – Scores respect intensity of each finding: 0 = <10%, 1 = 10-25%, 2 = 25-50%, 3 = > 50%.
