# Supplementary material for: Current treatment of lupus nephritis: an overview of the new guidelines
Source: J Bras Nefrol. 2025 Oct 13;47(4):e20250092. doi: 10.1590/2175-8239-JBN-2025-0092en (PMC12520630; doi:10.1590/2175-8239-JBN-2025-0092en)
Supplement: Table S3 - [file 2175-8239-jbn-47-4-e20250092-suppl3.pdf]

Braz. J. Nephrol.
https://doi.org/10.1590/2175-8239-JBN-2025-0092en

Supplementary Material to "Current treatment of lupus nephritis: an overview of the new guidelines"

Table S3 - Brazilian Society of Rheumatology principles and recommendations for the treatment of proliferative classes of lupus nephritis.

**General immunosuppression**
1. Hydroxychloroquine should be prescribed for all patients with SLE, unless contraindicated.
2. Glucocorticoids should be used at the lowest dose and for the shortest possible duration.
**Induction Therapy**
3. Initial induction therapy involves the use of MMF or intravenous CYC.
4. MMF and FK combination can be used in induction therapy, especially in the absence of response or limitation to CYC or MMF dose.
5. BEL and MMF combination can be used in induction therapy according to specific patient characteristics.
6. FK use as monotherapy can be used in induction therapy if MMF, CYC, MMF + FK, or BEL + MMF cannot be used.
7. MMF and voclosporin combination can be considered for induction therapy after voclosporin is approved by Brazilian regulatory agencies.
8. CsA as monotherapy is not recommended for induction therapy.
9. LFN as monotherapy is not recommended for induction therapy.
10. Monthly pulse therapy with glucocorticoids is not recommended during induction therapy.
**Maintenance therapy**
11. Both MMF and AZA can be used as maintenance therapy.
12. Calcineurin inhibitors (FK or CsA) can be used as maintenance therapy in patients who cannot use MMF or AZA.
13. LFN can be used as maintenance therapy in patients who cannot use MMF or AZA.
14. CYC is not recommended for maintenance therapy.

Abbreviations – SLE: Systemic Lupus Erythematosus; MMF: Mycophenolate Mofetil; CYC: Cyclophosphamide; FK: Tacrolimus; BEL: Belimumab; CsA: Cyclosporine A; LFN: Leflunomide; AZA: Azathioprine.
