# Supplementary material for: Current treatment of lupus nephritis: an overview of the new guidelines
Source: J Bras Nefrol. 2025 Oct 13;47(4):e20250092. doi: 10.1590/2175-8239-JBN-2025-0092en (PMC12520630; doi:10.1590/2175-8239-JBN-2025-0092en)
Supplement: Table S4 - [file 2175-8239-jbn-47-4-e20250092-suppl4.pdf]

Braz. J. Nephrol.
https://doi.org/10.1590/2175-8239-JBN-2025-0092en

Supplementary Material to "Current treatment of lupus nephritis: an overview of the new guidelines"

Table S4 - Induction therapy for class V lupus nephritis according to SBR.

**Induction therapy**	
CYC	Euro-Lupus regimen<br>NIH regimen
MMF	2-3 g/d for 6 months
AZA	2 mg/kg/d
Calcineurin inhibitors (FK or CsA) ± MMF	CsA 2.5 to 5 mg/kg/dia, FK 0.05 to 0.1mg/kg/dia divided in two doses with serum level adjustment in case of monotherapy. When using calcineurin inhibitors in combination with MMF, the usual prescription is MMF 1g/day + FK 4 mg/day.

Abbreviations – SBR: Brazilian Society of Rheumatology; CYC: Cyclophosphamide; NIH: National Institute of Health; MMF: Mycophenolate Mofetil; AZA: Azathioprine; FK: Tacrolimus; CsA: Cyclosporine A.
