# Supplementary material for: Current treatment of lupus nephritis: an overview of the new guidelines
Source: J Bras Nefrol. 2025 Oct 13;47(4):e20250092. doi: 10.1590/2175-8239-JBN-2025-0092en (PMC12520630; doi:10.1590/2175-8239-JBN-2025-0092en)
Supplement: Tabela S1 - [file 2175-8239-jbn-47-4-e20250092-suppl5.pdf]

Braz. J. Nephrol.
https://doi.org/10.1590/2175-8239-JBN-2025-0092pt

Material Suplementar para “Tratamento atual da nefrite lúpica: visão geral das novas diretrizes”

Tabela S1 – Classificação histológica da ISN/RPS 2003 para nefrite lúpica.

**Classe**	**Achados histopatológicos**
I – Mesangial mínima	Imunofluorescência com depósitos em mesângio e microscopia óptica normal
II – Mesangial proliferativa	Proliferação mesangial sem lesões endocapilares ou subepiteliais
III – Proliferativa focal	Proliferação endocapilar em menos de 50% dos glomérulos<br>Subclassificada em: ativa, ativa/crônica e crônica (A, A/C e C)
IV – Proliferativa difusa	Proliferação endocapilar em mais de 50% dos glomérulos<br>Subclassificada em: ativa, ativa/crônica e crônica (A, A/C e C) e em segmentar (lesões que acometem < 50% do glomérulo) ou global (> 50% do glomérulo) – S ou G
V – Membranosa	Presença de depósitos subepiteliais detectados por microscopia óptica, eletrônica ou imunofluorescência. Pode vir associada com outras classes.
VI – Esclerótica avançada	Presença de esclerose glomerular global de > 90% dos glomérulos e sem lesões ativas

Abreviações – ISN/RPS: Sociedade Internacional de Nefrologia / Sociedade de Patologia Renal.
