# Supplementary material for: Current treatment of lupus nephritis: an overview of the new guidelines
Source: J Bras Nefrol. 2025 Oct 13;47(4):e20250092. doi: 10.1590/2175-8239-JBN-2025-0092en (PMC12520630; doi:10.1590/2175-8239-JBN-2025-0092en)
Supplement: Tabela S2 - [file 2175-8239-jbn-47-4-e20250092-suppl6.pdf]

Braz. J. Nephrol.
https://doi.org/10.1590/2175-8239-JBN-2025-0092pt

Material Suplementar para "Tratamento atual da nefrite lúpica: visão geral das novas diretrizes"

Tabela S2 – Critérios que compõem os índices de atividade e cronicidade na avaliação da nefrite lúpica proliferativa.

Critérios de atividade	Pontos	Critérios de cronicidade	Pontos
Proliferação endocapilar	0-3	Glomérulos globalmente esclerosados	0-3
Infiltração leucocitária	0-3	Crescentes fibrosas/ fibrocelulares	0-3
Depósitos hialinos subendoteliais	0-3	Atrofia tubular	0-3
Necrose fibrinoide/cariorrexe (x2)	0-3	Fibrose intersticial	0-3
Crescentes celulares / fibrocelulares (x2)	0-3		
Inflamação intersticial	0-3		
**Total**	**0-24**	**Total**	**0-12**

Nota – Os pontos respeitam a intensidade de cada componente: 0 = <10%, 1 = 10-25%, 2 = 25-50%, 3 = > 50%.
