# Supplementary material for: Current treatment of lupus nephritis: an overview of the new guidelines
Source: J Bras Nefrol. 2025 Oct 13;47(4):e20250092. doi: 10.1590/2175-8239-JBN-2025-0092en (PMC12520630; doi:10.1590/2175-8239-JBN-2025-0092en)
Supplement: Tabela S3 - [file 2175-8239-jbn-47-4-e20250092-suppl7.pdf]

Braz. J. Nephrol.
https://doi.org/10.1590/2175-8239-JBN-2025-0092pt

Material Suplementar para “Tratamento atual da nefrite lúpica: visão geral das novas diretrizes”

Tabela S3 - Princípios e recomendações SBR para tratamento das classes proliferativas de NL.

**Imunossupressão geral**
1. Hidroxicloroquina deve ser prescrita para todos os pacientes com LES, exceto se contraindicado.
2. Glicocorticoides devem ser utilizados na menor dose e pelo menor período necessário.
**Terapia de indução**
3. A terapia de indução inicial envolve o uso de MMF ou CFF intravenosa.
4. A combinação de MMF e TAC pode ser usada na terapia de indução, especialmente na ausência de resposta ou limitação para MMF na dose de indução ou CFF .
5. A combinação de BEL e MMF pode ser utilizada na terapia de indução conforme características específicas do paciente.
6. O uso de TAC como imunossupressor em monoterapia pode ser utilizado na terapia de indução se MMF, CFF, MMF + TAC ou BEL + MMF não puderem ser usados.
7. A combinação de MMF e voclosporina pode ser considerada para terapia de indução após aprovação da voclosporina pelos órgãos reguladores brasileiros.
8. CsA como imunossupressor em monoterapia não é recomendada para terapia de indução.
9. LFN como imunossupressor em monoterapia não é recomendada para terapia de indução.
10. A terapia mensal de pulsoterapia com glicocorticoide não é recomendada durante a terapia de indução.
**Terapia de manutenção**
11. Tanto MMF quanto AZA podem ser usados como terapia de manutenção.
12. Inibidores da calcineurina (TAC ou CsA) podem ser utilizados como terapia de manutenção em pacientes que não podem usar MMF ou AZA.
13. LFN pode ser utilizada como terapia de manutenção em pacientes que não podem usar MMF ou AZA.
14. CFF não é recomendada para terapia de manutenção

Abreviações – SBR: Sociedade Brasileira de Reumatologia; NL: Nefrite Lúpica; LES: Lúpus Eritematoso Sistêmico; TAC: Tacrolimus; CsA: Ciclosporina A; MMF: Micofenolato de Mofetila; AZA: Azatioprina; CFF: Ciclofosfamida; BEL: Belimumabe; RTX: Rituximabe; LFN: Leflunomida. Nota – Baseada em tabela da SBR.
