# Supplementary material for: Current treatment of lupus nephritis: an overview of the new guidelines
Source: J Bras Nefrol. 2025 Oct 13;47(4):e20250092. doi: 10.1590/2175-8239-JBN-2025-0092en (PMC12520630; doi:10.1590/2175-8239-JBN-2025-0092en)
Supplement: Tabela S4 - [file 2175-8239-jbn-47-4-e20250092-suppl8.pdf]

Braz. J. Nephrol.
https://doi.org/10.1590/2175-8239-JBN-2025-0092pt

Material Suplementar para "Tratamento atual da nefrite lúpica: visão geral das novas diretrizes"

Tabela S4 - Terapia de indução para NL classe V pela SBR.

**Indução inicial NL V**	
CFF	Protocolo Euro-Lupus<br>Protocolo NIH
MMF	2-3 g/dia por 6 meses
AZA	2 mg/kg/dia
ICN (TAC ou CsA) ± MMF	CsA 2,5 a 5 mg/kg/dia, TAC 0,05 a 0,1 mg/kg/dia dividida em duas tomadas com ajuste de níveis séricos em caso de monoterapia.<br>Quando em uso de ICN associado ao MMF, são prescritos usualmente MMF 1 g/dia + TAC 4 mg/dia.

Abreviações – NL: Nefrite Lúpica; SBR: Sociedade Brasileira de Reumatologia; CFF: Ciclofosfamida ; MMF: Micofenolato de Mofetila; AZA: Azatioprina; ICN: inibidor de calcineurina; TAC: Tacrolimus; CsA: Ciclosporina A.
