# Supplementary material for: Current treatment of lupus nephritis: an overview of the new guidelines
Source: J Bras Nefrol. 2025 Oct 13;47(4):e20250092. doi: 10.1590/2175-8239-JBN-2025-0092en (PMC12520630; doi:10.1590/2175-8239-JBN-2025-0092en)
Supplement: Translation

# Translation: Tratamento atual da nefrite lúpica: visão geral das novas diretrizes

**DOI:** 10.1590/2175-8239-JBN-2025-0092pt

**Authors:** Gabriel Teixeira Montezuma Sales, Natália Janoni Macedo, Edgard Torres dos Reis, Gianna Mastroianni Kirsztajn

**Affiliations:** 1 Universidade Federal de São Paulo, Escola Paulista de Medicina, Departamento de Medicina, São Paulo, SP, Brasil.

**Keywords:** Nefrite Lúpica, Lúpus Eritematoso Sistêmico, Diretrizes, KDIGO, Imunossupressão, Glomerulopatias, Alvos Terapêuticos

## Abstract

**Introdução:** Face à relevância do acometimento renal no lúpus eritematoso sistêmico (LES) e às novas abordagens da doença e de seu tratamento, este artigo teve como objetivo sintetizar as principais atualizações no diagnóstico, manejo e tratamento da nefrite lúpica (NL), com base nas recentes publicações de diretrizes internacionais de referência em nefrologia e reumatologia, além de destacar aspectos de interesse das diretrizes nacionais da Sociedade Brasileira de Reumatologia (SBR) de 2024. São descritos os tratamentos por classes da nefrite lúpica, bem como os alvos terapêuticos, ressaltando semelhanças e diferenças entre as diretrizes. De modo geral, estas recomendam que o tratamento de indução (“inicial”) das classes proliferativas seja realizado com terapia isolada com micofenolato ou ciclofosfamida intravenosa, ou com esquemas multialvo, com corticoide, micofenolato ou ciclofosfamida e inibidor de calcineurina ou belimumabe como terceira droga. Deve-se considerar a mudança de terapia caso não se atinja o alvo de resposta esperado, que apresenta sutis diferenças entre os consensos atuais. O tratamento de manutenção (“subsequente”) deve ser realizado preferencialmente com micofenolato, azatioprina ou terapias multialvo. Novas evidências científicas trouxeram opções de tratamento com impacto no manejo da nefrite lúpica, justificando a publicação de novas diretrizes nos últimos meses; analisá-las de maneira crítica, poderá auxiliar na tomada de decisão para o tratamento individualizado das pessoas com a doença.

## Introdução

O Lúpus Eritematoso Sistêmico (LES) é uma doença inflamatória crônica autoimune, cuja etiopatogenia envolve a interação entre fatores genéticos e ambientais, caracterizando-se por ampla variabilidade de apresentação, curso clínico e gravidade. Em geral, cursa com períodos alternados de atividade e remissão, podendo acometer múltiplos órgãos e sistemas, sendo o rim um órgão frequentemente afetado. O acometimento renal no LES pode ocorrer em seus diversos compartimentos: túbulo-intersticial, vascular e glomerular, podendo apresentar-se como diferentes síndromes associadas às doenças glomerulares (síndrome nefrótica e nefrítica, perda rápida de função renal, alterações urinárias assintomáticas, entre outras)^1^.

A incidência de nefrite lúpica (NL) varia de forma considerável entre as diferentes regiões do mundo, raças e etnias, acometendo 20–60% das pessoas com LES, a depender da população estudada^2–5^. O acometimento renal no LES, por sua vez, está associado a um aumento da morbimortalidade, especialmente nos pacientes que progridem para falência renal – evento observado em cerca de 10–30% dos casos - percentual que pode ser superior naqueles que apresentam classes histológicas proliferativas^6–9^.

O principal objetivo do tratamento da NL é alcançar a remissão completa (RC), que está associada a bom prognóstico renal em longo prazo^8,10^. A sobrevida renal em 10 anos aumenta de 46% para 95% se a remissão for alcançada^11^. Contudo, apesar dos esquemas terapêuticos disponíveis, menos de 50% dos pacientes evolui com RC após os 6 primeiros meses do tratamento e, entre aqueles que atingem a remissão, observa-se ocorrência de recidivas em cerca de 25% dos pacientes dentro de 2–3 anos^10,12–14^.

Nesse sentido, o tratamento da NL tem sido alvo de numerosos estudos na última década, impulsionados pelo desenvolvimento de novas tecnologias e opções terapêuticas. Destacam-se, inclusive, estudos que demonstram a possibilidade de terapias multialvo como esquema de primeira linha para tratamentos de indução (“inicial”, segundo nomenclatura mais recentemente adotada) nas classes proliferativas, seja com belimumabe associado à ciclofosfamida (CFF) ou ao micofenolato (MMF), seja com inibidores de calcineurina (iCN), especialmente a voclosporina, associados ao MMF. Isso levou a discussões sobre uma possível “mudança de paradigma” no tratamento da NL, com a consideração do uso precoce de terapias combinadas^15–18^.

Diante da relevância do acometimento renal no LES e das novas abordagens da doença e de seu tratamento, este artigo de revisão teve como objetivo sintetizar as principais atualizações no diagnóstico e tratamento da NL, com base em três diretrizes internacionais de referência em nefrologia e reumatologia publicadas recentemente, KDIGO (*Kidney Disease: Improving Global Outcomes*) 2024, EULAR (*European League Against Rheumatism*) 2023 e ACR (*American College of Rheumatology*) 2025, além de destacar aspectos de interesse das diretrizes nacionais da Sociedade Brasileira de Reumatologia (SBR), publicadas em 2024^5,17,18^, com o intuito de auxiliar nefrologistas, reumatologistas e médicos em geral a se familiarizarem com as novas recomendações na área, visando alcançar a melhor prática para o tratamento da NL.

## Mecanismos Etiopatogênicos

A fisiopatologia da doença é complexa e heterogênea. O LES se desenvolve em indivíduos com susceptibilidade genética, levando à perda da tolerância imunológica, associada à exposição a determinados gatilhos ambientais que estimulam a resposta inflamatória, como infecções virais, exposição solar e alterações hormonais^19–22^. Somando-se a isso, há falha no processo de apoptose de neutrófilos - NETose -, que aumenta a exposição de antígenos nucleares ao sistema imune, contribuindo para o desenvolvimento de autoimunidade crônica^19,22,23^.

O acometimento renal no LES ocorre a partir da deposição de imunocomplexos (IC) circulantes no tecido renal, ou pela formação de IC *in situ*, de diferentes tamanhos, afinidades e cargas, o que explica a presença, em alguns casos, de depósitos mesangiais, subendoteliais e subepiteliais^24^. Esses IC, ao se depositarem no tecido renal promovem ativação da via clássica do complemento, de macrófagos e neutrófilos, o que também contribui para a amplificação da resposta imune inflamatória no local. Esse processo culmina em graus variáveis de lesão podocitária, proliferação celular mesangial, endotelial e do epitélio parietal, bem como aumento na síntese e deposição de matriz extracelular, levando à disfunção renal^20–22^.

## Quadro Clínico e Diagnóstico

O acometimento renal no LES pode permanecer assintomático por um longo período e, por esse motivo, os pacientes devem ser monitorados ativamente quanto à possibilidade de atividade renal. A avaliação deve incluir parâmetros clínicos e laboratoriais, envolvendo, pelo menos, análise do sedimento urinário, relação proteinúria/creatininúria e avaliação da função renal por meio da dosagem de creatinina sérica e do cálculo da taxa de filtração glomerular estimada (TFGe), realizada no mínimo no momento do diagnóstico e repetida a cada 6-12 meses, mesmo em pessoas com LES que sejam assintomáticas^5,25^.

Apesar de alterações no sedimento urinário e/ou na função renal constituírem importantes indícios de NL em atividade, os achados clínico-laboratoriais nem sempre se correlacionam com a extensão ou a gravidade do acometimento renal histológico^26,27^. Em geral, pacientes com sedimento urinário ativo e proteinúria > 0,5 g/24h - comumente acompanhada de hematúria microscópica e/ou leucocitúria -, especialmente se apresentarem hipocomplementemia e anti-DNA de dupla hélice (ds) positivo, exibem classes proliferativas de NL. Vale destacar que uma calculadora utilizando os parâmetros sedimento urinário ativo, creatinina sérica e anti-DNAds demonstrou boa acurácia na predição de classe proliferativa versus membranosa (https://nefritelupica.medicalcore.com.br)^28^. Porém, a biópsia renal, quando disponível, permanece como padrão-ouro para a definição da classe da NL e para o diagnóstico diferencial, bem como para graduar atividade e cronicidade, recursos que auxiliam na tomada de decisões terapêuticas e na avaliação do prognóstico renal^5^.

## Biópsia Renal em Pacientes com Lúpus Eritematoso Sistêmico

A Sociedade Internacional de Nefrologia e a Sociedade de Patologia Renal estabeleceram, em 2003, a classificação histológica atualmente utilizada para a NL, a qual é dividida em seis classes (conforme Tabela S1), baseadas nos achados de microscopia óptica e imunofluorescência. Segundo essas normatizações, devem ser avaliados os glomérulos, os túbulos e o interstício, com a descrição de índices de atividade e cronicidade, bem como o compartimento vascular, que pode evidenciar condições associadas, como a síndrome do anticorpo antifosfolípide (SAAF) e outras microangiopatias trombóticas, com uma prevalência estimada de até 24%^29^.

O tipo mais comum e mais grave é a glomerulonefrite proliferativa, que pode ser focal (classe III) ou difusa (classe IV). Além disso, é possível observar mais de uma classe histológica durante a evolução da doença ou até mesmo de forma simultânea^30,31^. A presença de classes proliferativas (III e IV) associadas à classe V representa um fator de mau prognóstico, devendo ser tratada de forma agressiva com regimes imunussopressores^9,30^. Em alguns casos, a microscopia eletrônica pode também ser útil para o diagnóstico, permitindo, por exemplo, confirmar uma podocitopatia lúpica - definida histologicamente pela retração difusa dos processos podocitários, mas sem depósitos imunes ao longo das alças capilares -, o que a diferencia de outras formas de NL, com uma prevalência estimada de até 2%^31,32^.

Além de definir a classe da NL, a biópsia renal também possibilita a quantificação dos índices de atividade e cronicidade, sendo o primeiro graduado de 0 a 24 pontos e o segundo de 0 a 12 pontos, conforme descrito na Tabela S2. Embora não exista uma definição clara nas diretrizes, pacientes com lesões predominantemente crônicas provavelmente não necessitam de imunossupressão para a NL, mas podem se beneficiar da utilização de medidas antiproteinúricas, por exemplo^25^.

Diante da suspeita de abertura de um quadro de atividade renal, recomenda-se que a biópsia seja realizada sempre que possível, uma vez que a determinação da classificação da NL com base apenas em parâmetros clínicos e laboratoriais é limitada. Tanto a EULAR quanto o KDIGO recomendam a realização de biópsia renal na presença de proteinúria ≥ 0,5 g/24horas ou em casos de piora da função renal sem outra causa conhecida^5,17^.

A indicação de rebiópsia ainda é motivo de controvérsias, e a repetição da biópsia renal diante de recidivas de atividade renal nem sempre é necessária^33^. Contudo, dados emergentes sugerem que biópsias seriadas podem auxiliar na tomada de decisões de tratamento em curso e na predição do prognóstico renal. A realização de biópsias repetidas demonstrou discordância considerável entre a atividade da doença clínica e histológica^25^. Estudos evidenciaram que, após 6 a 8 meses de terapia imunossupressora, 20% a 50% do total de pacientes com critérios para remissão clínica ainda apresentavam evidência histológica de inflamação ativa, enquanto 40% a 60% dos pacientes sem evidência histológica de atividade da doença ainda mantinham proteinúria persistente de alto grau^26,34^.

Em relação à resposta renal em longo prazo, foram descritas evidências de atividade histológica em cerca de 20% dos pacientes com remissão clínica sustentada, mesmo após anos de tratamento imunossupressor, enquanto 40% dos pacientes com proteinúria residual mantida apresentavam remissão histológica completa^35^. Esses achados evidenciam um possível papel da rebiópsia como ferramenta auxiliar na decisão sobre a retirada ou a continuidade da terapia de manutenção (“subsequente”, segundo a nomenclatura mais recentemente adotada), embora não haja, até o momento, recomendação formal nas diretrizes quanto a essa prática^17,25^. As indicações de biópsia e rebiópsia estão resumidas na Tabela 1.

**Tabela 1:** Resumo das indicações de biópsia renal em pacientes com lúpus eritematoso sistêmico

1^a^Biópsia – realizar sempre que possível, quando:	
Proteinúria ≥ 0,5 g/24h (acompanhada ou não de outros comemorativos) Elevação da creatinina sérica sem outra causa aparente	
**Rebiópsia – possíveis indicações:**	**Observações**
Auxílio na decisão sobre a retirada da terapia de manutenção (após pelo menos 3 anos, incluindo as fases de indução e manutenção) Recidiva de atividade renal para reavaliação da classe, especialmente se apresentação inicial com classe não proliferativa. NL refratária à terapia de indução, geralmente após 24 semanas, se houver suspeita de outros diagnósticos associados ou evolução para cronicidade. É necessário individualizar.	Atividade histológica mantida, mesmo com resposta clínica completa, sugere manter imunossupressão de manutenção.

## Tratamento da Nefrite Lúpica

O diagnóstico e consequente início precoce do tratamento são pontos essenciais na terapia da NL, uma vez que, em suas fases iniciais, a doença tende a ser oligossintomática e pode progredir para doença renal crônica em poucos meses. Por se tratar de uma doença crônica, que apresenta alto risco de recidiva, o tratamento imunossupressor na NL geralmente inclui tanto uma fase de indução quanto uma fase de manutenção, com o objetivo de evitar novas agudizações e manter a remissão^17^.

### Aspectos Gerais do Tratamento

Além do uso de imunossupressores, diversas outras medidas são importantes, incluindo fotoproteção (uso de filtro solar com fator de proteção ≥ 30, preferencialmente > 50, e limitação da área de exposição), medidas adjuvantes para redução de progressão para doença renal crônica, controle dos fatores de risco cardiovasculares, prevenção de infecções, planejamento familiar e contracepção, bem como avaliação da saúde óssea^5,17^. A seguir, abordaremos algumas das principais medidas gerais de tratamento, lembrando que nossos objetivos de tratamento devem ir muito além da simples indução de remissão (Figura 1).

**Figura 1.** Objetivos do tratamento do paciente com nefrite lúpica. Adaptada de Echavarria et al.^84^

#### Risco Cardiovascular

As complicações cardiovasculares são uma causa importante de morbimortalidade em pessoas com LES, devido a fatores de risco tradicionais e não tradicionais. Além disso, o uso de corticoides e inibidores de calcineurina pode levar a eventos adversos, como hipertensão arterial sistêmica (HAS), disglicemia, dislipidemia e outros aspectos relacionados ao aumento do risco cardiovascular. Dessa forma, além do controle da atividade da doença, a avaliação regular dos fatores de risco cardiovasculares tradicionais e a instituição de medidas para reduzi-los, como modificações no estilo de vida (cessação do tabagismo, controle do peso e prática regular de atividade física), bem como o controle e tratamento da glicemia, da HAS e da dislipidemia, são essenciais para a prevenção de eventos cardiovasculares^5,17^.

#### Risco de Infecções

Trata-se, atualmente, de uma das principais causas de óbito nessa população, especialmente no período inicial do tratamento, quando a imunossupressão é mais intensa^36,37^. Nesse sentido, devem ser implementadas estratégias de prevenção para mitigar esse risco. O uso de sulfametoxazol/trimetoprim não é formalmente recomendado pelas diretrizes analisadas, diferentemente do que ocorre em outras doenças, como as vasculites ANCA-associadas, sobretudo devido à ocorrência de reações adversas à sulfa em um número considerável de casos. Entretanto, alguns estudos sugerem a profilaxia em pacientes que usam imunossupressores (CFF ou MMF), prednisona em dose maior ou igual a 20 mg por período superior a 4 semanas, nefrite em atividade e/ou doença intersticial pulmonar^38^.

A atualização da carteira vacinal, de acordo com a faixa etária e a condição de imunossupressão, deve ser sempre estimulada, preferencialmente quando a doença estiver inativa e antes do início de qualquer terapia imunossupressora. As vacinas inativadas, bem como as vacinas de subunidade, peptídicas ou proteicas e genéticas são seguras em qualquer fase do tratamento, enquanto as vacinas com microrganismos atenuados devem ser evitadas na vigência de imunossupressão^5^. Recomenda-se que sejam realizadas as imunizações contra infecções, incluindo influenza, pneumocócica, meningocócica, COVID-19, *Herpes zoster* (vacina recombinante inativada) e HPV^5,17^.

#### Doença Renal Crônica (DRC)

Como em qualquer outra causa de DRC, as medidas para retardar a sua progressão são de extrema importância na NL, incluindo controle pressórico adequado, evitar dieta hiperproteica e com excesso de sódio, cessação do tabagismo e manutenção da prática regular de exercícios físicos. Quanto às medidas farmacológicas específicas, recomenda-se o uso do bloqueio do sistema renina-angiotensina (inibidores da enzima conversora de angiotensina [IECA] ou bloqueadores do receptor de angiotensina [BRA]) nos casos de pacientes com HAS ou com proteinúria > 300 mg/24h (sugerido se > 30 mg/24h). Os inibidores do co-transportador sódio-glicose 2 (iSGLT2) podem ser considerados em associação aos IECA ou BRA, se TFGe > 20 mL/min/1,73m^2^, especialmente se houver albuminúria > 0,2 g/g (creatinina urinária), embora ainda faltem estudos prospectivos que demonstrem o benefício dessa medicação especificamente na população com NL^39^.

#### Saúde Óssea

A terapia com glicocorticoides, especialmente quando em doses ≥ 5 mg/dia por mais de três meses, acelera a perda de massa óssea. Dessa forma, a suplementação de cálcio (ingestão ideal de 1.000 a 1.200 mg/d), quando necessária, e de vitamina D, com o objetivo de manter nível sérico entre 30 e 60 ng/mL, é recomendada para pacientes em uso de corticoides. O uso de medicações antirreabsortivas ou anabólicas deve ser considerado de acordo com a avaliação individual do risco de fratura, o qual pode ser estimado com base em dados demográficos, no histórico clínico do paciente, na pontuação no escore FRAX (Ferramenta de Avaliação de Risco de Fratura) e na osteoporose induzida pelo glicocorticoide^5^.

#### Contracepção

A gravidez em pessoas com NL está associada a piores desfechos materno-fetais em comparação à população saudável. Além disso, algumas medicações empregadas no tratamento do LES, como MMF, CFF, metotrexate e leflunomida, são contraindicadas durante a gestação. Dessa forma, todas as mulheres em idade fértil devem receber aconselhamento sobre gestação e contracepção, a fim de identificar possíveis contraindicações e definir o momento mais adequado para engravidar^5^. Atualmente, recomenda-se que a gestação seja planejada para um período de pelo menos 6 meses de doença controlada, sem uso de medicações contraindicadas durante a gravidez. Os métodos anticoncepcionais que contêm estrogênio devem ser evitados em pacientes com doença em atividade, história prévia de evento cardiovascular ou risco aumentado para a ocorrência de fenômenos tromboembólicos, como nos casos de SAAF^40,41^.

#### Hidroxicloroquina

Ambas as diretrizes atualizadas do KDIGO e da EULAR recomendam que todos os pacientes com LES recebam tratamento com hidroxicloroquina (HCQ), com grau de recomendação 1, a menos que haja alguma contraindicação^5,17^. A HCQ apresenta múltiplos benefícios para pessoas com LES, por meio de efeitos diretos e indiretos, incluindo: melhora da atividade da doença e redução de recidivas; melhora das lesões de pele e de queixas articulares; redução das doses cumulativas de corticoides; potencialização da ação de outras medicações, como o MMF; prevenção de dano; melhora do perfil lipídico e da glicemia; redução do risco de síndrome metabólica e de fenômenos trombóticos; além da diminuição do risco de mortalidade. Esta classe de medicamentos já é utilizada no tratamento do LES há mais de 50 anos e, dentre as drogas antimaláricas, deve-se dar preferência à HCQ em relação ao difosfato de cloroquina, devido ao seu melhor perfil de segurança^17,42^.

Os antimaláricos são melanotrópicos e concentram-se em estruturas oculares que contêm melanina, como o epitélio pigmentado da retina e a coroide. O seu acúmulo na retina pode afetar as células fotorreceptoras, especialmente os cones da mácula, região central da retina responsável pela visão. O risco de toxicidade retiniana é maior com o difosfato de cloroquina do que com a HCQ. É importante ressaltar que as lesões oculares e o estabelecimento do dano podem ser assintomáticos, e os sintomas, quando presentes, podem surgir tardiamente. Os principais sintomas incluem: diminuição da acuidade visual, alterações na percepção de cores, manchas ou áreas de perda de visão (escotomas) e visão turva^43^.

Atualmente, a dose recomendada de HCQ é de 5 mg/kg/dia, com dose máxima de 400mg/dia. Para pacientes com TFGe < 30 mL/min/1,73m^2^, a dose deve ser reduzida em 30-50%^5,44–46^. Nas doses recomendadas, o risco de toxicidade é inferior a 1% em até 5 anos de uso e inferior a 2% em até 10 anos; porém, aumenta para quase 20% após 20 anos de uso da medicação. Após esse período, mesmo o paciente sem toxicidade prévia apresenta 4% de risco no ano subsequente. Os principais fatores de risco para toxicidade ocular são dose diária acima da recomendada, tempo de uso, insuficiência renal, maculopatia ou retinopatia prévias e uso concomitante de tamoxifeno. Outros fatores de risco incluem: idade avançada, insuficiência hepática e fatores genéticos possivelmente relacionados a anormalidades do gene ABCA4 ou no citocromo P450^47^.

Em 2016, a Sociedade Americana de Oftalmologia atualizou suas recomendações para o rastreamento da retinopatia em pacientes em uso de difosfato de cloroquina ou HCQ. Recomenda-se que pacientes que iniciarão a droga sejam submetidos a exame oftalmológico dentro do primeiro ano de tratamento, incluindo o exame de fundo de olho. O exame de campo visual e a tomografia de coerência óptica de domínio espectral (OCT-SD), embora úteis no início do tratamento, não são obrigatórios, a menos que o paciente apresente fatores de risco ou outras doenças (por exemplo, maculopatia prévia, glaucoma ou outras) que possam afetar os testes de rastreamento inicial. Na ausência dos principais fatores de risco, os exames de rastreamento podem ser realizados anualmente a partir de cinco anos após a avaliação inicial. Se fatores de risco estiverem presentes, exames de rastreamento devem ser realizados anualmente, sendo recomendadas a avaliação automatizada de campo visual e a OCT-SD. Em algumas situações, exames adicionais podem ser indicados, como o eletroretinograma multifocal (mfERG), que fornece informações objetivas sobre o campo visual, e a autofluorescência de fundo (FAF), capaz de evidenciar topograficamente o dano, especialmente em pacientes asiáticos^47^. OCT-SD e campo visual são técnicas mais sensíveis, com potencial para detecção precoce de alterações relacionadas ao uso da HCQ. Vale ressaltar que não se deve esperar a alteração típica de *bull’s eye maculopathy* para o diagnóstico da toxicidade aos antimaláricos, uma vez que este achado é tardio e irreversível^48^.

### Tratamento da Nefrite Lúpica Classes i e ii e da Podocitopatia Lúpica

Pacientes com as classes mesangiais de NL (I e II) geralmente apresentam uma evolução benigna, com hematúria microscópica e proteinúria de baixo grau. Nesses casos, o início de imunossupressão específica não é necessário, a menos que haja outras lesões de órgão-alvo que demandem tratamento imunossupressor^49^.

Segundo as diretrizes do KDIGO para nefrite lúpica, nos casos de pacientes que apresentem padrão histológico de classes I ou II à microscopia de luz, mas manifestem clinicamente síndrome nefrótica, deve-se aventar a hipótese de podocitopatia lúpica. O diagnóstico pode ser confirmado por microscopia eletrônica, que evidencia retração difusa dos processos podocitários, na ausência de depósitos subendoteliais ou subepiteliais. Nesses casos, a doença costuma apresentar o mesmo comportamento das podocitopatias primárias (doença de lesão mínima/ glomeruloesclerose segmentar e focal) e deve ser manejada de forma semelhante, utilizando terapia com glicocorticoides, geralmente apresentando boa resposta^32,50,51^. Contudo, na podocitopatia lúpica, há uma aparente maior tendência à recidiva, embora os dados sejam escassos. O tratamento de manutenção deve ser considerado nesse contexto, apesar de o tempo e o agente ideais não estarem bem definidos. Como opções, pode-se considerar a associação de glicocorticoides em dose baixa com MMF, azatioprina ou iCN^5,52^. A EULAR não traz recomendações específicas para as classes I e II.

### Tratamento Da Nefrite Lúpica Classes iii e iv

As classes proliferativas são as mais frequentemente diagnosticadas e as que apresentam maior chance de evolução com perda de função renal, apresentando prevalência de DRC estágio V de até 30% em 10 anos^53^. Nas últimas décadas, o tratamento dessas classes passou por grandes mudanças: do uso isolado de corticoides até a década de 1970, para o uso de CFF associado aos corticoides até os anos 2000, resultando em impacto importante na sobrevida renal e geral de pessoas com LES. No início dos anos 2000, foram publicados os primeiros estudos com MMF como uma alternativa eficiente e com potencial para determinar menos efeitos adversos. Dessa forma, nas últimas décadas, o tratamento de indução de primeira linha da NL restringiu-se ao uso de MMF (2–3 g/dia de micofenolato de mofetila) ou CFF intravenosa, seja na dose de 500 mg a cada 2 semanas por 3 meses (protocolo do *Euro-Lupus Nephritis Trial*), seja em doses mensais de 0,5–1,0 g/m^2^ de área de superfície corporal por 6 meses (protocolo do *National Institutes of Health* [NIH], USA)^54–56^. Vale ressaltar que, no presente texto, o termo “micofenolato (MMF)” pode representar micofenolato de mofetila ou micofenolato de sódio, sendo frequentemente referidas as doses do primeiro, embora ambos sejam utilizados na prática clínica.

Curiosamente, após cerca de 20 anos sem a aprovação de novas terapias, surgiram, nos últimos anos, diversos *trials* randomizados demonstrando novas opções de tratamento^5^. Em 2020, foi publicado o estudo BLISS-LN, com avaliação do belimumabe - anticorpo monoclonal que inibe o BAFF (fator ativador de linfócitos B) - associado ou não à terapia padrão (CFF Euro-Lupus ou MMF) na NL classes III ou IV(±V) ou classe V ativa. O estudo incluiu 448 pacientes randomizados, acompanhados por 2 anos^57^. Uma maior proporção de pacientes alcançou remissão completa, assim como resposta renal primária eficaz, particularmente em pacientes com uma relação proteína/creatinina urinária inicial inferior a 3 g/g. Além disso, a adição do belimumabe esteve associada a maior preservação da função renal e menor quantidade de recidivas^57–59^.

Em 2021, o estudo AURORA 1, com voclosporina (inibidor de calcineurina mais potente, com menor incidência de eventos adversos que a ciclosporina e o tacrolimus e sem a necessidade de acompanhamento de nível sérico), associada ao MMF e prednisona em baixas doses, demonstrou maior chance de alcançar remissão completa em comparação ao MMF e prednisona^60^. Em 2015, um estudo em população oriental com tratamento multialvo (micofenolato de mofetila 1g/dia e tacrolimus 4 mg/dia), já havia demonstrado benefício da associação de inibidor de calcineurina na indução de pacientes com NL proliferativa; entretanto, devido à restrição étnica do estudo, esses resultados não foram suficientes para justificar a inclusão dessa classe de medicamentos como primeira linha de tratamento^61^. Mais recentemente, o AURORA 2, com acompanhamento de 3 anos, demonstrou manutenção de uma maior taxa de remissão, sem piora da função renal dos pacientes^62^.

Diante da maior taxa de remissão, sem aumento do risco de eventos adversos, e da exiguidade de opções terapêuticas com bom perfil de segurança disponíveis, tanto o belimumabe quanto a voclosporina foram aprovados em diversos países como opções de tratamento para NL classes III ou IV (±V), sempre em associação à terapia padrão. Com base em todas essas novas evidências, tanto as diretrizes do KDIGO quanto as diretrizes americanas, europeias e brasileiras de reumatologia divulgaram atualizações com novas recomendações para o tratamento da NL classes III e IV (±V).

A EULAR e o KDIGO descrevem quatro esquemas de primeira linha para o tratamento das classes proliferativas, associados à prednisona e hidroxicloroquina, conforme listado abaixo (Tabela 2)^5,17^.

**Tabela 2:** Esquemas de 1^a^ linha para tratamento das classes proliferativas, conforme eular e kdigo

Opções para terapia de indução de 1^a^ linha nas classes proliferativas	Observações
Terapia isolada com ciclofosfamida	Preferência por dose reduzida de 500 mg a cada 14 dias por 3 meses, correspondendo ao esquema Euro-Lupus
Terapia isolada com micofenolato de mofetila	Dose de 2–3 g/dia
Belimumabe em associação	Dose de 10 mg/kg IV nos D0, D14, D28 e a cada 28 dias Com micofenolato ou ciclofosfamida Não indicado se TFGe < 30 ml/min/1,73m^2^
Voclosporina associada ao micofenolato de mofetila 2g/dia	Alternativas: micofenolato de mofetila 1g + tacrolimus (0,05–0,1 mg/dia, dividido em 2 tomadas, com ajuste por nível sérico) ou ciclosporina (3 mg/kg/dia, dividido em 2 tomadas, com ajuste por nível sérico), na ausência da voclosporina. Não indicado se TFGe< 45 ml/min/1,73m^2^

Existem diversas semelhanças entre ambas as diretrizes internacionais. Em nenhuma delas há uma hierarquia clara entre os esquemas, sendo necessário avaliar o risco de má adesão, o perfil de efeitos adversos e as manifestações extrarrenais no processo de decisão. Em pacientes com proteinúria nefrótica, dá-se preferência ao uso de inibidores de calcineurina, enquanto a terapia com belimumabe demonstrou menor eficácia nesta população. Ambas ponderam sobre a importância da queda da proteinúria e da manutenção/melhora da função renal como parâmetros precoces de avaliação da resposta renal, considerando a queda > 25% da proteinúria em até 3 meses um sinal de boa resposta clínica, associada à estabilidade da função renal, com redução máxima da TFGe de 20% em relação ao valor basal. Além disso, enfatizam a importância do uso de imunossupressores por pelo menos 3 anos, com sua retirada realizada preferencialmente de forma lenta e progressiva.

Quanto ao uso de corticoides, há consenso de que estes devem ser os primeiros imunossupressores a serem retirados, com cautela e redução lenta e progressiva, em razão do risco de recidiva^63^. Atualmente, observa-se também uma tendência à utilização de doses reduzidas de corticoides na terapia de indução. Sugere-se o uso de pulsoterapia com metilprednisolona, em doses de 125 a 500 mg, por 1 a 3 dias, além da introdução de doses mais baixas e com redução mais rápida da dose do corticoide oral, visando atingir doses ≤ 5 mg/dia em 3 a 6 meses. Essa tendência é particularmente aplicável a pacientes menos graves e que estejam evoluindo de forma favorável, uma vez que doses reduzidas apresentam resposta semelhante com menor risco de eventos adversos^60^.

Em relação a diversos outros pontos, não há concordância entre ambas diretrizes. Em casos mais graves, o KDIGO não considera necessária qualquer alteração no tratamento, limitando-se a citar que a associação com belimumabe pode ser vista como opção para pacientes com maior risco de evolução para DRC, como aqueles que já se apresentam com alteração da função renal ou com altos índices de atividade e/ou cronicidade. A EULAR propõe que se reserve o uso da CFF em altas doses, conforme o protocolo do NIH, para pacientes com elevado risco de disfunção renal, especialmente aqueles que já apresentam disfunção renal grave e/ou que, na biópsia, evidenciem crescentes celulares, necrose fibrinoide e/ou inflamação intersticial grave. O protocolo Euro-Lupus apresenta-se como uma opção preferível para os demais casos, por sua menor dose cumulativa e menor toxicidade associada. Já o KDIGO traz ambos os protocolos endovenosos como opções iniciais, ressaltando que o protocolo do Euro-Lupus foi testado em população predominantemente caucasiana, enquanto o estudo do NIH envolveu uma população multiétnica. O KDIGO também menciona a CFF oral como alternativa, com a mesma eficácia, embora implique um esquema com maior dose cumulativa de CFF e toxicidade.

Apesar da ausência de descrição de uma hierarquia no KDIGO entre os tratamentos, pondera-se dar preferência ao uso de esquemas que incluam belimumabe em pacientes com histórico de recidivas, enquanto o inibidor da calcineurina deve ser priorizado em casos de intolerância a doses elevadas de MMF ou na presença de proteinúria > 3g/24h, como citado anteriormente. Os esquemas multialvo também podem ser considerados se houver dificuldade na diminuição da dose de prednisona.

Em relação ao tratamento de manutenção, o KDIGO recomenda o MMF como primeira escolha para a maioria dos pacientes, excetuando-se, principalmente, aqueles que desejam engravidar - situação em que se dá preferência à azatioprina. Já a EULAR considera que o tratamento de manutenção deve ser escolhido de acordo com a indução: pacientes inicialmente tratados com CFF isolada poderiam utilizar tanto azatioprina quanto MMF e aqueles que receberam indução com MMF manteriam o mesmo fármaco. Ambas sugerem que os pacientes submetidos à terapia com três drogas devem manter este tratamento por um período de 3 anos.

### Tratamento da Nefrite Lúpica Classe v

A incidência de NL classe V isolada varia de 5–20%. Essa forma pode associar-se a classes proliferativas e está relacionada a um maior risco de síndrome nefrótica e trombose de veia renal^5,64–66^. O risco de perda de função renal está associado à gravidade da proteinúria e, diferentemente do que se descreve na glomerulopatia membranosa primária, não costuma cursar com remissão espontânea^65,67,68^. Dessa forma, a NL classe V configura uma forma potencialmente grave, e a decisão acerca do tratamento deve ser realizada com base principalmente no grau de proteinúria, mas também na presença de perda progressiva da função renal.

Considerando o nível de proteinúria como critério para indicação de tratamento, observa-se uma discordância entre as duas diretrizes. A EULAR recomenda o uso de imunossupressor em casos de proteinúria persistente > 1g/24h, enquanto o KDIGO orienta iniciar a imunossupressão especialmente em pacientes com síndrome nefrótica^5,17^. Nesse caso, a falta de consenso é explicada sobretudo por amostragens menores de classe V pura nos estudos randomizados. Por esse motivo, sugerimos individualizar o tratamento nos pacientes com proteinúria > 1g/24h, considerando a estabilidade e a gravidade da proteinúria, a função renal, além dos riscos e benefícios do tratamento e a atividade da doença em outros órgãos e sistemas^5^.

Em relação ao regime imunossupressor a ser utilizado, não há evidência de alta qualidade que oriente a recomendação para NL classe V sem componente proliferativo associado. Um pequeno ensaio clínico randomizado demonstrou maior taxa de remissão com a associação de CFF com corticosteroide, bem como com iCN e corticosteroide, quando comparada ao uso de corticosteroide isolado, embora com maior taxa de recidiva nos pacientes que receberam iCN em relação à CFF^69^. Além de CFF e iCN, outros estudos pequenos também avaliaram a eficácia do MMF, azatioprina e rituximabe (RTX), em combinação com corticoterapia, com taxas de resposta variando entre 40–60%^5^.

No estudo AURORA, em que 14% dos pacientes apresentavam NL classe V isolada, a adição de voclosporina à terapia padrão (MMF e corticoide) mostrou-se mais efetiva do que a imunossupressão padrão isolada em alcançar resposta renal em 31 pacientes com NL classe V. Ao analisar outro desfecho, embora sem significância estatística, observou-se menor tempo médio de redução da proteinúria para 0,5 mg/mg em pacientes tratados com voclosporina em associação com corticoide e MMF (3,6 meses), em comparação aos controles tratados com corticoide, MMF e placebo (8,3 meses)^60^. Em relação ao belimumabe, resultados da análise *post-hoc* do estudo BLISS-LN (16% com NL classe V pura) sugeriram que pacientes com proteinúria nefrótica não apresentaram benefício no desfecho primário, embora tenha sido observada uma tendência à redução de outros desfechos, como perda de > 30% da TFGe e diminuição do número de recidivas^59^.

Apesar da ausência de recomendações baseadas em evidências robustas, o KDIGO 2024 é favorável à utilização de MMF, iCN, azatioprina, RTX ou o uso por curto período de CFF^5^. Já a EULAR recomenda o MMF como primeira escolha, colocando o iCN e a CFF como opções alternativas e reservando o RTX para pacientes com NL classe V refratária^70^. Em 2023, a Sociedade Espanhola de Nefrologia publicou uma diretriz que individualizou um pouco mais a conduta, sugerindo um esquema com iCN e MMF para pacientes com síndrome nefrótica. Para os pacientes sem síndrome nefrótica, mas com proteinúria > 1 g/24h, a diretriz recomenda a administração de iCN^71^.

### Critérios de Resposta e Alvo Terapêutico

A avaliação da resposta à terapia baseia-se na melhora da proteinúria e na estabilização ou melhora da função renal. Entretanto, não existe um critério universalmente aceito quanto ao nível de melhora necessário, o que dificulta a comparação direta entre diferentes ensaios clínicos^5^. Análises *post-hoc* de dois grandes estudos randomizados europeus indicaram que os níveis de proteinúria após 12 meses de tratamento são o melhor preditor de desfechos renais a longo prazo, tendo encontrado melhora desses desfechos (risco de doença renal crônica em estágio terminal ou duplicação da creatinina sérica em 10 anos) quando níveis de proteinúria < 0,7–0,8 g/24h eram alcançados^72–76^. A EULAR, portanto, reforça que a terapia deve ter como objetivo um alvo de proteinúria <0,5–0,7g/24h em até 12 meses, embora até 50% dos pacientes que não atinjam esse alvo também possam manter função renal estável no longo prazo^70,73,77^.

A análise do estudo ALMS sugeriu que a redução de 25% dos níveis de proteinúria e a normalização dos níveis de complemento em 8 semanas de tratamento predizem desfechos renais favoráveis. Esse achado serviu de base para que a EULAR considerasse o corte de queda da proteinúria > 25% como meta de tratamento (no caso em 3 meses), em conjunto com melhora/estabilização da função renal, e queda da proteinúria > 50% em 6 meses^70,78,79^. Os alvos de proteinúria para avaliação da resposta à terapia durante o primeiro ano do tratamento de indução estão sintetizados na Tabela 3.

**Tabela 3:** Fatores a serem considerados na escolha da terapia imunossupressora na prática clínica

	MMF	CFF NIH	CFF Euro-Lupus	AZA	TAC	CsA	BEL	RTX
Favorece adesão		X	X					
Facilidade de acesso	X	X	X	X		X		
Necessidade de centro de infusão		X	X				X	X
Gravidade ou refratariedade		X					X	X
Gestação/lactação				X	X	X		
Risco de infertilidade		X	X					
Alto custo					X		X	X

Abreviaturas – TAC: Tacrolimus; CsA: Ciclosporina A; MMF: Micofenolato de Mofetila; AZA: Azatioprina; CFF: Ciclofosfamida; BEL: Belimumabe; RTX: Rituximabe. Nota – Adaptado de tabela da SBR.

Ainda no que se refere à resposta ao tratamento (Tabela 4), o KDIGO ressalta que a melhora da proteinúria e da TFGe ocorrem de forma contínua ao longo do tempo, com a taxa de melhora variando consideravelmente entre os pacientes. Além disso, existem diferenças marcantes entre a proteinúria e a TFGe basal no momento da apresentação da doença, o que também influencia o tempo de resposta. Proteinúrias em faixa nefrótica, por exemplo, podem levar um período maior que 6-12 meses para normalização ou redução^5,80^. Apesar de essa diretriz também enfatizar o bom prognóstico associado à queda da proteinúria > 25% em 3 meses e < 0,7-0,8 g/24h em 12 meses, especialmente em casos de proteinúria nefrótica, não é estritamente necessário que esses marcos temporais sejam atingidos para a tomada de decisões, sendo aceitável aguardar um período maior (de 18 a 24 meses) para a obtenção de uma resposta completa em pacientes que estejam apresentando melhoria contínua, conforme o KDIGO^5^.

**Tabela 4:** Critérios para definição dos tipos de resposta ao tratamento na nefrite lúpica, conforme as diretrizes do kdigo 2024

Tipos de resposta	Definição
Remissão completa	Redução da proteinúria para <0,5 g/g de P/C ou proteinúria de 24h Estabilização ou melhora da função renal (valor dentro de ±10%–15% da TFGe basal) Em 6 a 12 meses após o início da terapia
Resposta renal de eficácia primária	P/C < 0,7 g/g Redução máxima da TFGe de 20% do valor basal ou TFGe > 60 ml/min/1,73 m^2^ Sem uso de terapia de resgate Associada a melhores desfechos renais
Remissão parcial	Redução da proteinúria em pelo menos 50% e para <3 g/g de P/C ou proteinúria de 24h Estabilização ou melhora da função renal (±10%–15% do valor basal) Em 6 a 12 meses após o início da terapia
Ausência de resposta	Sem critérios de RC ou RP após 6–12 meses de ISS

Abreviaturas – ISS: Imunossupressão; P/C: Relação Proteína/Creatinina na Urina; RC: Remissão Completa; RP: Remissão Parcial; TFGe: Taxa de Filtração Glomerular estimada. Nota – Adaptado de tabela do KDIGO.

### Nefrite Lúpica Refratária

A avaliação de resposta tradicionalmente é feita em 8 a 12 semanas, mas, se o paciente não apresentar qualquer redução da proteinúria e/ou melhora da função renal em 4 semanas, o prognóstico é considerado desfavorável, e a investigação de possíveis causas de refratariedade já deve ser abordada.

O primeiro passo recomendado pelo KDIGO nos casos refratários é a avaliação da adesão ao tratamento, considerando, nesses casos, o uso de CFF (por ser endovenoso). Alguns estudos com avaliação de nível sérico, especialmente com hidroxicloroquina, têm mostrado uma correlação direta entre níveis séricos com maior incidência de recidiva, ressaltando a importância tanto da avaliação da adesão quanto da variabilidade da dose interpessoal^81^.

Caso seja confirmada a adesão, recomenda-se considerar nova biópsia renal para avaliar a possibilidade de achados que justifiquem a falta de resposta, como a presença de altos índices de cronicidade e de microangiopatia trombótica. Se confirmada a presença de atividade renal reversível na vigência do tratamento otimizado, a recomendação é a troca para um esquema de primeira linha diferente do inicial.

Por fim, na ausência de resposta aos esquemas disponíveis, devem ser considerados tratamentos de segunda linha, como o RTX, doses altas estendidas de CFF, além do recrutamento em estudos randomizados com novas medicações^5^.

Ressaltamos que, além das medicações atualmente recomendadas pelas diretrizes do KDIGO e da EULAR, uma nova opção tem se mostrado promissora no tratamento de indução da nefrite lúpica: o obinutuzumabe. Trata-se de um anticorpo monoclonal humanizado anti-CD20, cuja eficácia foi recentemente avaliada no estudo REGENCY, um ensaio clínico de fase 3, em associação à terapia padrão com micofenolato de mofetila. O desfecho primário avaliado foi a taxa de resposta renal completa na semana 76, definida como relação proteína/creatinina urinária < 0,5 e TFGe ≥ 85% do valor basal. Como resultado, 46,4% dos pacientes no grupo que recebeu obinutuzumabe alcançaram resposta renal completa, em comparação com 33,1% no grupo placebo. Apesar dos achados favoráveis, são necessários estudos adicionais para respaldar a incorporação formal do obinutuzumabe como opção terapêutica para pacientes com nefrite lúpica ativa^82^.

## Destaques do Consenso da Sociedade Brasileira de Reumatologia Para o Diagnóstico e Tratamento da Nefrite Lúpica

Além dos principais pontos trazidos pelas diretrizes internacionais, também é válido mencionar as principais recomendações em relação ao manejo da NL na realidade brasileira. Nesse sentido, a SBR publicou, em 2024, atualizações nas recomendações de tratamento da NL, com 14 recomendações (Tabela S3) elaboradas segundo a metodologia GRADE, levando-se em consideração o acesso e a disponibilidade de medicações em âmbito nacional. O documento apresenta ainda uma proposta de fluxograma de tratamento e sugestões para a prática clínica. Ademais, diversos fatores inerentes ao paciente também devem ser avaliados no momento da escolha da terapia. Entre aqueles que podem auxiliar na tomada de decisão em relação à terapia imunossupressora (Tabela 3), destacam-se: adesão do paciente à medicação, disponibilidade, acesso, necessidade de centro de infusão, gravidade ou refratariedade, recidivas prévias, compatibilidade com gestação/lactação, risco de infertilidade e custos das medicações.

O fluxograma da SBR sugere iniciar o tratamento de indução das classes proliferativas com terapia isolada com MMF ou CFF intravenosa. Em concordância com a EULAR, o fluxograma da SBR também reforça a necessidade de alcançar a resposta renal alvo, que deve ser frequentemente reavaliada no primeiro ano do tratamento (aos 3 meses, 6 meses e ao final de 1 ano, conforme descrito na Tabela 5). Na ausência de resposta ao esquema inicial, sugere-se a troca do esquema ou a realização de associações, conforme descrito na Figura 2. O tacrolimus em monoterapia também é opção para os casos em que as demais drogas não possam ser utilizadas. Em situações de falha terapêutica com pelo menos duas terapias de indução ou contraindicação aos tratamentos padronizados, após a revisão de fatores como adesão ao fármaco, dose da medicação imunossupressora e comorbidades concomitantes, além da consideração da necessidade de rebiópsia, está indicado o uso de RTX. O tratamento de indução inicial sugerido para NL classe V também está descrito na Tabela S4. Para a escolha da medicação, deve-se considerar a gravidade do quadro, conforme os níveis de proteinúria e de albumina sérica, além dos fatores individuais do paciente citados anteriormente.

**Tabela 5:** Alvos de proteinúria no 1o ano do tratamento

Resposta renal alvo – proteinúria
Redução ≥ 25% em 3 meses
Redução ≥ 50% em 6 meses
Redução para < 0,7–0,8 g/24h em 12 meses

**Figura 2.** Terapia de indução para classes proliferativas de NL pela SBR. Adaptado de Reis-Neto et al.^18^

Abreviaturas – TAC: Tacrolimus; MMF: Micofenolato de Mofetila; AZA: Azatioprina; CFF: Ciclofosfamida; BEL: Belimumabe; RTX: Rituximabe; NIH: protocolo do *National Institutes of Health*.

Uma vez que no Brasil não há possibilidade de acesso universal à biópsia renal, o consenso da SBR ressalta que, quando a biópsia não estiver disponível ou houver alguma contraindicação ao procedimento, a decisão terapêutica pode ser ancorada em parâmetros clínicos e laboratoriais, já que o atraso na instituição do tratamento imunossupressor (principalmente nos casos suspeitos de glomerulonefrite rapidamente progressiva) está associado a pior prognóstico renal em curto e longo prazo^25^.

O Consenso de NL da SBR destaca ainda a importância das medidas adjuvantes à terapia imunossupressora para uma boa resposta ao tratamento, incluindo fotoproteção, informação e educação do paciente, controle da pressão arterial, uso de medicações antiproteinúricas, avaliação do risco cardiovascular e controle da proteinúria, tratamento da osteoporose induzida por corticoides, investigação de anticorpos antifosfolipídeos e prevenção de tromboembolismo, imunização e prevenção de infecções, além de evitar o tabagismo e drogas nefrotóxicas.

## Destaques do Consenso do Colégio Americano de Reumatologia Para o Diagnóstico e Tratamento da Nefrite Lúpica.

Complementando a análise das principais diretrizes vigentes sobre o manejo da nefrite lúpica, vale destacar aspectos relevantes do Consenso do ACR, publicado em maio de 2025. Este documento apresenta algumas recomendações terapêuticas que divergem daquelas previamente estabelecidas por outras sociedades e que foram aqui descritas, no que se refere à terapia de indução da NL, conforme sumarizado na Figura 3 ^83^.

**Figura 3.** Comparação entre as diretrizes do tratamento de primeira linha da nefrite lúpica proliferativa.

Abreviaturas – MMF: Micofenolato de Mofetila; CFF: Ciclofosfamida; iCN: inibidor de Calcineurina; NIH: protocolo do *National Institutes of Health*; DRC: Doença Renal Crônica; TFGe: Taxa de Filtração Glomerular estimada

O ACR recomenda como esquema de primeira linha para o tratamento da NL proliferativa apenas esquemas com terapia tripla: belimumabe ou inibidor de calcineurina associado a micofenolato e prednisona, considerando que os resultados recentes dos estudos AURORA e BLISS-LN 2 demonstraram superioridade desses esquemas, justificando a diferença de hierarquia entre as terapias triplas em comparação às terapias duplas^59,60,62^. No processo de decisão entre belimumabe e iCN, alguns pontos foram considerados pelo consenso: 1) o belimumabe deve ser priorizado em pacientes com TFGe < 45 ml/min/1,73m^2^, com elevado índice de cronicidade ou com pressão arterial > 165 × 105 mmHg, devido à nefrotoxicidade e ao descontrole pressórico associados aos iCN; 2) pacientes com manifestações extrarrenais devem ser considerados prioritários para o uso do belimumabe, em razão de sua ação benéfica, especialmente no que se refere aos acometimentos cutâneo e articular; 3) pacientes com proteinúria ≥ 3g/g (se TFGe > 45 ml/min/1,73m^2^) devem utilizar iCN, uma vez que não se demonstrou benefício do belimumabe, em comparação aos esquemas duplos, em induzir remissão nesses pacientes^59,83^.

Outro ponto de destaque do consenso foi o posicionamento de priorizar o uso do micofenolato em vez da ciclofosfamida, considerando o menor risco de malignidade e de infertilidade do primeiro, embora ainda admita a ciclofosfamida como uma boa opção em pacientes com intolerância gástrica ao micofenolato, histórico de má aderência ou que se apresentem com glomerulonefrite rapidamente progressiva e/ou necrose fibrinoide. O consenso também enfatiza a importância de minimizar o uso de corticoides, estabelecendo como limite máximo, após pulsoterapia, a dose de 0,5 mg/kg/dia ou 40 mg/dia.

Quanto às demais classes, devem-se enfatizar as recomendações para a classe V, nas quais a proteinúria também é considerada determinante para a escolha do tratamento. Recomenda-se a terapia tripla com iCN, MMF e corticoide como primeira linha em pacientes com proteinúria > 1g/24h, enquanto MMF, corticoide, azatioprina ou iCN isoladamente são as opções indicadas para pacientes com proteinúria < 1g/24h.

## Conclusões

O manejo da NL é uma das áreas da nefrologia que vem passando por grandes mudanças nos últimos anos, especialmente em relação às opções de tratamento de indução, o que justifica a publicação, pelas sociedades de reumatologia e de nefrologia, de novas recomendações de tratamento nos últimos meses. Embora utilizem os mesmos estudos como base, essas diretrizes apresentam alguns pontos de vista diferentes. Conhecer suas semelhanças e diferenças poderá auxiliar na tomada de decisão para o tratamento individualizado de nossos pacientes.

## Data Availability

Todo o conjunto de dados que dá suporte ao texto deste artigo de revisão encontra-se disponível e pode ser acessado a partir das referências bibliográficas citadas.

## Material Suplementar

O seguinte material online está disponível para o presente artigo:

Tabela S1 - Classificação histológica da ISN/RPS 2003 para nefrite lúpica.

Tabela S2 - Critérios que compõem os índices de atividade e cronicidade na avaliação da nefrite lúpica proliferativa.

Tabela S3 - Princípios e recomendações da SBR para tratamento das classes proliferativas de NL.

Tabela S4 - Terapia de indução para NL classe V pela SBR.
